# Supplementary material for: Using machine learning to predict risk of incident opioid use disorder among fee-for-service Medicare beneficiaries: A prognostic study
Source: PLoS One. 2020 Jul 17;15(7):e0235981. doi: 10.1371/journal.pone.0235981 (PMC7367453; doi:10.1371/journal.pone.0235981)
Supplement: S1 Appendix — (DOCX) [file pone.0235981.s001.docx]

**Appendix A. Compliance to the 2015 Standards for Reporting Diagnostic Accuracy (STARD) checklist**

| **Section & Topic** | **No** | **Item** | **Reported on page #** |
| --- | --- | --- | --- |
| **TITLE OR ABSTRACT** |  |  |  |
|  | **1** | Identification as a study of diagnostic accuracy using at least one measure of accuracy (such as sensitivity, specificity, predictive values, or AUC) | Abstract (Page 1) |
| **ABSTRACT** |  |  |  |
|  | **2** | Structured summary of study design, methods, results, and conclusions  (for specific guidance, see STARD for Abstracts) | Pages 5-6 |
| **INTRODUCTION** |  |  |  |
|  | **3** | Scientific and clinical background, including the intended use and clinical role of the index test | Page 7 |
|  | **4** | Study objectives and hypotheses | Page 8 |
| **METHODS** |  |  |  |
| ***Study design*** | **5** | Whether data collection was planned before the index test and reference standard  were performed (prospective study) or after (retrospective study) | Page 9 |
| ***Participants*** | **6** | Eligibility criteria | Page 9,10 |
|  | **7** | On what basis potentially eligible participants were identified  (such as symptoms, results from previous tests, inclusion in registry) | Page 9,10 |
|  | **8** | Where and when potentially eligible participants were identified (setting, location and dates) | Page 9,10 |
|  | **9** | Whether participants formed a consecutive, random or convenience series | Page 10 |
| ***Test methods*** | **10a** | Index test, in sufficient detail to allow replication | Pages 10-13 |
|  | **10b** | Reference standard, in sufficient detail to allow replication | Pages 10-13, and Appendix Methods |
|  | **11** | Rationale for choosing the reference standard (if alternatives exist) | Pages 10-13 |
|  | **12a** | Definition of and rationale for test positivity cut-offs or result categories  of the index test, distinguishing pre-specified from exploratory | Page 12, and Appendix Methods |
|  | **12b** | Definition of and rationale for test positivity cut-offs or result categories  of the reference standard, distinguishing pre-specified from exploratory | Page 12, and Appendix Methods |
|  | **13a** | Whether clinical information and reference standard results were available  to the performers/readers of the index test | Page 10-11 (Outcome variable) |
|  | **13b** | Whether clinical information and index test results were available  to the assessors of the reference standard | Pages 10-12 |
| ***Analysis*** | **14** | Methods for estimating or comparing measures of diagnostic accuracy | Pages 10-13, and Appendix Methods |
|  | **15** | How indeterminate index test or reference standard results were handled | Pages 10-11 |
|  | **16** | How missing data on the index test and reference standard were handled | Appendix Methods |
|  | **17** | Any analyses of variability in diagnostic accuracy, distinguishing pre-specified from exploratory | Page 10-13 |
|  | **18** | Intended sample size and how it was determined | Not available (due to use of existing data) |
| **RESULTS** |  |  |  |
| ***Participants*** | **19** | Flow of participants, using a diagram | eFigure 1 |
|  | **20** | Baseline demographic and clinical characteristics of participants | Page 13, Table 1 |
|  | **21a** | Distribution of severity of disease in those with the target condition | Page 14-15 |
|  | **21b** | Distribution of alternative diagnoses in those without the target condition | Page 14-15 |
|  | **22** | Time interval and any clinical interventions between index test and reference standard | Page 15 |
| ***Test results*** | **23** | Cross tabulation of the index test results (or their distribution)  by the results of the reference standard | eTables 5-7 |
|  | **24** | Estimates of diagnostic accuracy and their precision (such as 95% confidence intervals) | Page 13, Figure 1A-D eTable 5-6; eFigure 4-5 |

| **Section & Topic** | **No** | **Item** | **Reported on page #** |
| --- | --- | --- | --- |
|  | **25** | Any adverse events from performing the index test or the reference standard | Not applicable due to using existing data |
| **DISCUSSION** |  |  |  |
|  | **26** | Study limitations, including sources of potential bias, statistical uncertainty, and generalisability | Page 19 |
|  | **27** | Implications for practice, including the intended use and clinical role of the index test | Pages 18-19 |
| **OTHER INFORMATION** |  |  |  |
|  | **28** | Registration number and name of registry | Not applicable due to using existing data and analysed retrospectively |
|  | **29** | Where the full study protocol can be accessed | Pages 13, and Appendix Methods |
|  | **30** | Sources of funding and other support; role of funders | 2, and Acknowledgement (page 19) |
|  |  |  |  |
