# Supplementary material for: Using machine learning to predict risk of incident opioid use disorder among fee-for-service Medicare beneficiaries: A prognostic study
Source: PLoS One. 2020 Jul 17;15(7):e0235981. doi: 10.1371/journal.pone.0235981 (PMC7367453; doi:10.1371/journal.pone.0235981)
Supplement: S1 Text — (DOCX) [file pone.0235981.s003.docx]

**Appendix methods**

**Introduction**

In this study, our primary goal was prediction, and the secondary goal was risk stratification (i.e., to identify subgroups of patients at similar risk of the outcome). First, we randomly and equally divided beneficiaries into training, testing, and validation samples based on the characteristic and opioid use disorder (OUD) distribution. We developed and tested prediction algorithms for incident OUD using four machine learning approaches: elastic net (EN), random forests (RF), gradient boosting machine (GBM), and deep neural network (DNN). For each approach, we fit the trained algorithms based on the training sample, refined the algorithm using the testing sample, and then applied the final algorithm in the validation sample to evaluate prediction performance.

Our model reporting complies with the Standards for Reporting Diagnostic Accuracy (STARD) and the Transparent Reporting of Multivariable Prediction Model for Individual Prognosis or Diagnosis (TRIPOD) reporting guidelines.[1, 2] We calculated the C-statistic (or area under the receiver operating curve [ROC]) from the validation sample to assess discrimination (i.e., the extent to which patients predicted as high-risk exhibit higher OUD rates compared to those predicted as low risk). We examined any difference in C-statistics across different approaches using the DeLong Test.[3] For each probability cutoff point, OUD was predicted for the visits with calculated probabilities above the cutoff point, whereas non-OUD was predicted for the visits with probabilities below the cutoff points. Based on their true and predicted OUD status, the patients’ 3-month visits can be assigned to one of the four groups (i.e., true positive [TP], false positive [FP], true negative [TN], false negative [FN]) shown in the classification matrix (**S3 Fig**). Given that OUD events are rare outcomes and C-statistics do not incorporate information about the prevalence of the outcome, we reported other more appropriate metrics, including sensitivity, specificity, positive predictive value (PPV), negative predictive value (NPV), positive likelihood ratio (PLR), negative likelihood ratio (NLR), number needed to evaluate (NNE) to identify one OUD, and estimated rate of alerts to assess pre-implementation evaluation of our prediction algorithms (**S3 Fig**).[4] The optimal algorithm for a screening test depends on pre-test probability of the outcome, the values of TPs and TNs, and the costs of FP and FN. Since these factors vary from setting to setting (and some of them are subjective choices), no single cutoff point is suitable for every purpose. In order to compare performance across methods, we presented and assessed these prediction metrics (e.g., NNE) at the optimized threshold of the predicted probability that balances sensitivity and specificity as identified by the Youden index,[5] as well as at multiple levels of sensitivity and specificity (e.g., 90%-100%) to allow risk-benefit evaluations of interventions triggered by positive tests using different thresholds defining high risk.

Second, based on the individual’s predicted probability of an OUD event, we classified beneficiaries in the validation sample into decile risk subgroups, with the highest decile further split into three additional strata based on the top 1^st^, 2^nd^ to 5^th^, and 6^th^ to 10^th^ percentiles to allow closer examination of patients at highest risk of developing OUD. We evaluated calibration plots (the extent to which the predicted OUD risk agreed with the observed risks) by the risk subgroup. We briefly summarized our machine learning approaches in the sections below (see details in our previously published work).[6]

**Regularized logistic regression: Elastic net (EN)[7, 8]**

We chose to use EN regularization because it minimizes overfitting through parameter shrinkage and variable selection to create a parsimonious algorithm. It has been shown that EN outperforms and is more efficiently than traditional least absolute shrinkage and selection operator (LASSO) regularization.[8] Briefly, after forming a prediction model with logistic regression using all candidate variables, beta coefficients are penalized and lowered to deal with model overfitting. The magnitude of penalization is subsequently changed to create various models with different prediction errors in a cross-validation process, so the final model achieves optimal penalization based on the lowest prediction error. In regularized regression, variable selection is performed automatically by shrinking regression coefficients of some variables to zero. Specifically, the loss function in the EN regularization included log likelihood and regularization parameters λ_1_ and λ_2_ (smaller value indicating less penalization). The EN procedure generated a total of 40 candidate values for λ_1_ and λ_2_ from the training set. We used 5-fold cross-validation and used the 1-standard error (1-SE) rule to select the optimal λ_1_ and λ_2_ in the final model in the training and testing samples. We standardized continuous variables to improve optimization and convergence of the models. Similar to traditional statistical methods (e.g., logistic regression), regularized regression methods cannot handle missing values and they delete rows with missing data. For variables with missing information, we imputed with the median for continuous variables, and with the most frequent category for categorical variables. Our candidate model contained all predictor candidates, quadratic transformation of non-normal distributed continuous variables, and two-way interaction between two predictors. Inclusion of quadratic transformations is commonly practiced in conducting EN regularization to accommodate potential non-linear relationships between key candidate predictors and outcomes. Regularized regression is expected to be more effective in the following situations: (1) there are many more columns (predictors) than rows (observations), (2) when the predictors available may be extremely highly correlated with each other, or (3) the goal is to find the most compact model yielding an acceptable performance. We used the sklearn package in the Python 3.6 to perform EN. All other parameters were set as default values. For EN regularized regression, individuals in the validation sample were assigned to one of the two predictive categories (i.e., OUD vs. non-OUD) if the probability threshold was >0.42 (optimized threshold identified using the Youden Index), >0.95 (using top 1 percentile of predicted scores in the training sample), >0.77 (using the top 5 percentile of the predicted scores), and >0.61 (using the top 10 percentile of the predicted scores).

**Tree-structured approaches: Random Forests (RF)[9, 10] and Gradient Boosting Machine (GBM; Stochastic gradient boosting or TreeNet in Salford SPM)[11, 12]**

This study used two tree ensemble approaches including RF and GBM. An RF consists of a collection of trees grown in parallel, while GBM consists of a series of trees grown in a sequential order of successive trees to minimize residual error. For RF, at each split in a tree, a random sample of predictors is chosen. We followed the steps and rationale from the implementation of Chirkov et al.’s RF framework.[13] Prior to conducting RF, for variables with missing information, we imputed with the median for continuous variables, and with the most frequent category for categorical variables. We used the “*Random Forests Tree Ensembles”* in the software package Salford SPM for this study. The final fine-tuning parameters included the number of trees to build as 200, the number of predictor candidates randomly selected at each node as $\sqrt{number of total predictors}=\sqrt{269}$, using the balanced (i.e., upweight small classes to equal the size of the largest target class) class weight function, and an out of bag (OOB) function while other parameters remained as default. For RF, validation visits were assigned to one of the two predictive categories (i.e., OUD vs. non-OUD) if the probability threshold was >0.62 which was identified from the ROC using the Youden Index.

For GBM, we used the Salford’s TreeNet function to supply an initial value specific to the chosen loss function (i.e. logistic binary) for each record in the training sample. TreeNet can handle missing values automatically. We used cross entropy (i.e., negative average log likelihood) as the tuning criterion to determine the number of trees optimal for logistic models. Second, TreeNet sampled 25% of the records in the training sample randomly and then computed the generalized residual for the records in the sample. The first tree is fitted to the data and begins with a very small tree as the initial model. TreeNet used the sampled records to fit a classification tree with a maximum 8 terminal nodes to the generalized residuals. Third, TreeNet used the classification tree derived from the sampled records to update the TreeNet model based on the loss function and shrink the updated tree by the learning rate (or shrinkage rate) at 0.1 for overfitting protection. TreeNet repeated the steps previously described 200 times (i.e., number of trees to build = 200). Finally, we tested and validated the algorithms in the testing and validation samples. For TreeNet, validation visits were assigned to one of the two predictive categories (i.e., OUD vs. non-OUD) if the probability threshold >0.49 that was identified from the ROC using the Youden Index.

**Deep feed-forward neural network (DNN) or Artificial neural network (ANN)[14, 15]**

Deep learning refers to artificial neural network (ANN) models with multiple hidden layers (typically ≥3) that can be used to quantify the complex nonlinear relationships between inputs and outputs. Prior to conducting deep learning, we imputed missing data using median imputation for continuous variables and mode for categorical data. We also generated a series of dummy variables for categorical variables, and normalized continuous variables ranging between 0 and 1. We used a deep feed-forward neural network (DNN) to develop algorithms in our training datasets using Python 3.6 (*keras* package). We examined different numbers of hidden layers and nodes. At the end, the DNN with 2 hidden layers with 120 and 40 nodes performed the best. In this study, we generally referred to our approach as DNN although the final model only has two hidden layers. In each hidden layer during the algorithm-training process, we chose ReLU as an activation function to better prevent the vanishing gradient and to yield a faster convergence. We applied a sigmoid function to the output layer to generate a score representing the probability of OUD. We used the binary cross-entropy loss function with balanced class weight to adjust for the rare outcome. Finally, we conducted a hyperparameter search using the grid: L1 and L2 regularization weight. For DNN, validation visits were assigned to one of the two predictive categories (i.e., OUD vs. non-OUD) if the probability threshold >0.40 that was identified from the ROC using the Youden Index.

**References**

1. Collins GS, Reitsma JB, Altman DG, Moons KG. Transparent Reporting of a multivariable prediction model for Individual Prognosis or Diagnosis (TRIPOD): the TRIPOD statement. Ann Intern Med. 2015;162(1):55-63. Epub 2015/01/07. doi: 10.7326/M14-0697. PubMed PMID: 25560714.

2. Bossuyt PM, Reitsma JB, Bruns DE, Gatsonis CA, Glasziou PP, Irwig L, et al. STARD 2015: an updated list of essential items for reporting diagnostic accuracy studies. BMJ. 2015;351:h5527. Epub 2015/10/30. doi: 10.1136/bmj.h5527. PubMed PMID: 26511519; PubMed Central PMCID: PMCPMC4623764.

3. DeLong ER, DeLong DM, Clarke-Pearson DL. Comparing the areas under two or more correlated receiver operating characteristic curves: a nonparametric approach. Biometrics. 1988;44(3):837-45. Epub 1988/09/01. PubMed PMID: 3203132.

4. Romero-Brufau S, Huddleston JM, Escobar GJ, Liebow M. Why the C-statistic is not informative to evaluate early warning scores and what metrics to use. Crit Care. 2015;19:285. Epub 2015/08/14. doi: 10.1186/s13054-015-0999-1. PubMed PMID: 26268570; PubMed Central PMCID: PMCPMC4535737.

5. Fluss R, Faraggi D, Reiser B. Estimation of the Youden Index and its associated cutoff point. Biom J. 2005;47(4):458-72. Epub 2005/09/16. PubMed PMID: 16161804.

6. Lo-Ciganic WH, Huang JL, Zhang HH, Weiss JC, Wu Y, Kwoh CK, et al. Evaluation of Machine-Learning Algorithms for Predicting Opioid Overdose Risk Among Medicare Beneficiaries With Opioid Prescriptions. JAMA Netw Open. 2019;2(3):e190968. Epub 2019/03/23. doi: 10.1001/jamanetworkopen.2019.0968. PubMed PMID: 30901048.

7. Hastie T, Tibshirani R, Friedman J. The Elements of Statistical Learning: : Data Mining, Inference, and Prediction. 2nd ed. New York, NY: Springer; 2008.

8. Zou H, Hastie T. Regularization and variable selection via the elastic net. J R Stat Soc Series B Stat Methodol. 2005;67(Part 2):301-20.

9. Breiman L. Random Forest. Machine Learning. 2001;45:5-32.

10. Boulesteix AL, Janitza S, Kruppa J, Konig I. Overview of random forest methodology and practical guidance with emphasis on computational biology and bioinformatics. Wiley Interdisciplinary Reviews: Data Mining and Knowledge Discovery. 2012;2(6):493-507.

11. Friedman JH. Greedy Function Approximation: A Gradient Boosting Machine. Technical report, Dept. of Statistics, Stanford University. 1999.

12. Friedman JH. A Gradient Boosting Machine. Annals of statistics. 2001;29(5):1189.

13. Chirikov VV, Shaya FT, Onukwugha E, Mullins CD, dosReis S, Howell CD. Tree-based Claims Algorithm for Measuring Pretreatment Quality of Care in Medicare Disabled Hepatitis C Patients. Med Care. 2015. doi: 10.1097/MLR.0000000000000405. PubMed PMID: 26225448.

14. LeCun Y, Bengio Y, Hinton G. Deep learning. Nature. 2015;521(7553):436-44. Epub 2015/05/29. doi: 10.1038/nature14539. PubMed PMID: 26017442.

15. Schmidhuber J. Deep learning in neural networks: an overview. Neural Netw. 2015;61:85-117. Epub 2014/12/03. doi: 10.1016/j.neunet.2014.09.003. PubMed PMID: 25462637.

16. Bohnert AS, Valenstein M, Bair MJ, Ganoczy D, McCarthy JF, Ilgen MA, et al. Association between opioid prescribing patterns and opioid overdose-related deaths. JAMA. 2011;305(13):1315-21. Epub 2011/04/07. doi: 10.1001/jama.2011.370. PubMed PMID: 21467284.

17. Bohnert ASB, Valenstein M, Bair MJ, Ganoczy D, McCarthy JF, Ilgen MA, et al. Association between opioid prescribing patterns and opioid overdose-related deaths. JAMA. 2011;305(13):1315-21. doi: 10.1001/jama.2011.370. PubMed PMID: 21467284.

18. Centers for Medicare and Medicaid Services (CMS), “CY 2019 Final Call Letter,” [cited 2018 Nov 6]. Available from: <https://www.cms.gov/Medicare/Health-Plans/MedicareAdvtgSpecRateStats/Downloads/Announcement2019.pdf>.

19. Saito T, Rehmsmeier M. The precision-recall plot is more informative than the ROC plot when evaluating binary classifiers on imbalanced datasets. PLoS One. 2015;10(3):e0118432. Epub 2015/03/05. doi: 10.1371/journal.pone.0118432. PubMed PMID: 25738806; PubMed Central PMCID: PMCPMC4349800.
