# Supplementary material for: Using machine learning to predict risk of incident opioid use disorder among fee-for-service Medicare beneficiaries: A prognostic study
Source: PLoS One. 2020 Jul 17;15(7):e0235981. doi: 10.1371/journal.pone.0235981 (PMC7367453; doi:10.1371/journal.pone.0235981)
Supplement: S1 Table — (DOCX) [file pone.0235981.s004.docx]

**S1 Table. Diagnosis codes for the exclusion of patients with malignant cancers based on the National Committee for Quality Assurance (NCQA)’s Opioid Measures in 2018 Healthcare Effectiveness Data and Information Set (HEDIS)**

| **ICD-9 codes** | **ICD-10 codes** |
| --- | --- |
| 140.x (x=0, 1, 3, 4, 5, 6, 8, 9), 141.x (x=0, to 6, 8 and 9), 142.x (x=0, 1, 2, 8, 9), 143.x (x=0, 1, 8, 9), 144.x (x=0, 1, 8, 9), 145.x (x=0 to 6, 8, and 9), 146.x (x=0 to 9), 147.x (x=0, 1, 2, 3, 8, 9), 148.x (x=0, 1,2, 3, 8, 9), 149.x (x=0, 1, 8, 9), 150.x (x=0, 1, 2, 3, 4, 5, 8, 9), 151.x (x=0, 1, 2, 3, 4, 5, 6. 8, 9), 152.x (x=0, 1, 2, 3, 8, 9), 153.x (x=0 to 9), 154.x (x=0, 1, 2, 3, 8), 155.x (x=0, 1, 2), 156.x (x=0, 1, 2, 8, 9), 156.x (x=1, 2, 8, 9), 157.x (x=0, 1, 2, 3, 4, 8, 9), 158.x (x=0, 8, 9), 159.x (x=0, 1, 8, 9), 160.x (x=0 to 5, 8, 9), 161.x (x=0, 1, 2, 3, 8, 9), 162.x (x=0, 2, 3, 4, 5, 8, 9), 163.x (x=0, 1, 8, 9), 164.x (x=0, 1, 2, 3, 8, 9), 165.x (x=0, 8, 9), 170.x (x=0 to 9), 171.x (x=0, 2, 3, 4, 5, 6, 7, 8, 9), 172.x (x=0 to 9), 174.x (x=0 to 6, 8, 9), 175.0, 175.9, 176.x (x=0 to 5, 8, 9), 179, 180.x (x=0, 1, 8, 9), 181, 182.x (x=0, 1, 8), 183.x (x=0, 2, 3, 4, 5, 8, 9), 184.x (x=0, 1, 2, 3, 4, 8, 9), 185, 186.0, 186.9, 187.x (x=1 to 9), 188.x (x=0 to 9), 189.x (x=0, 1, 2, 3, 4, 8, 9), 190.x (x=0 to 9), 191.x (x=0 to 9), 192.x (x=0, 1, 2, 3, 8, 9), 193, 194.x (x=0, 1, 3, 4, 5, 6. 8, 9), 195.x (x=0 to 5, 8), 196.x (x=0 to 3, 5, 6, 8, 9), 197.x (x=0 to 8), 198.x (x=0 to 7), 198.81, 198.82, 198.89, 199.x (x=0, 1, 2), 200.0x (x=0 to 8), 200.1x (x=0 to 8), 200.2x (x=0 to 8), 200.3x (x=0 to 8), 200.4x (x=0 to 8), 200.5x (x=0 to 8), 200.6x (x=0 to 8), 200.7x (x=0 to 8), 200.8x (x=0 to 8), 201.0x (x=0 to 8), 201.1x (x=0 to 8), 201.2x (x=0 to 8), 201.4x (x=0 to 8), 201.5x (x=0 to 8), 201.6x (x=0 to 8), 201.7x (x=0 to 8), 201.9x (x=0 to 8), 202.0x (x=0 to 8), 202.1x (x=0 to 8), 202.2x (x=0 to 8), 202.3x (x=0 to 8), 202.4x (x=0 to 8), 202.5x (x=0 to 8), 202.6x (x=0 to 8), 202.7x (x=0 to 8), 202.8x (x=0 to 8), 202.9x (x=0 to 8), 203.0x (x=0 to 2), 203.1x (x=0 to 2), 203.8x (x=0 to 2), 204.0x (x=0 to 2), 204.1x (x=0 to 2), 204.2x (x=0 to 2), 204.8x (x=0 to 2), 204.9x (x=0 to 2), 205.0x (x=0 to 2), 205.1x (x=0 to 2), 205.2x (x=0 to 2), 205.3x (x=0 to 2), 205.8x (x=0 to 2), 205.9x (x=0 to 2), 206.0x (x=0 to 2), 206.1x (x=0 to 2), 206.2x (x=0 to 2), 206.8x (x=0 to 2), 206.9x (x=0 to 2), 207.0x (x=0 to 2), 207.1x (x=0 to 2), 207.2x (x=0 to 2), 207.8x (x=0 to 2), 208.0x (x=0 to 2), 208.1x (x=0 to 2), 208.2x (x=0 to 2), 208.8x (x=0 to 2), 208.9x (x=0 to 2), 209.0x (x=0 to 3), 209.1x (x=0 to 7), 209.2x (x=0 to 7, 9), 209.3x (x=0 to 6), 209.7x (x=0 to 5, 9). | C00.x, C01, C02.x (x=0, 1, 2, 3, 4, 8, 9), C03.x (x=0, 1, 9), C04.0,  C04.1, C04.8, C04.9, C05.0, C05.1, C05.2, C05.8, C05.9, C06.0, C06.1,  C06.2, C06.80, C06.89, C06.9, C07, C08.0, C08.1, C08.9, C09.0, C09.1,  C09.8, C09.9, C10.x (x=0, 1, 2, 3, 4, 8, 9), C11.x (x=0, 1, 2, 3, 8, 9), C12, C13.x (x=0, 1, 2, 3, 8, 9), C14.0, C14.2, C14.8, C15.3, C15.4, C15.5, C15.8, C15.9, C16.x (x=0, 1, 2, 3, 4, 5, 6, 8, 9), C17.x (x=0, 1, 2, 3, 8, 9),  C18.x, C19, C20, C21.x (x=0, 1, 2, 8), C22.x (x=0, 1, 2, 3, 4, 7, 8, 9), C23, C24.x (x=0, 1, 8, 9), C24.1, C24.8, C24.9, C25.x (x=0, 1, 2, 3, 4, 7, 8, 9), C26.0, C26.1, C26.9, C30.0, C30.1, C31.x (x=0, 1, 2, 3, 8, 9), C32.x (x=0, 1, 2, 3, 8, 9), C33, C34.00, C34.01, C34.02, C34.10, C34.11, C34.12, C34.2, C34.30, C34.31, C34.32, C34.80, C34.81, C34.82, C34.90, C34.91, C34.92, C37, C38.x (x=0, 1, 2, 3, 4, 8), C39.0, C39.9, C40.x0 (x=0, 1, 2, 3, 8, 9), C40.x1 (x=0, 1, 2, 3, 8, 9), C40.02 (x=0, 1, 2, 3, 8, 9), C41.x (x=0, 1, 2, 3, 4, 9), C43.0, C43.10, C43.11, C43.12, C43.20, C43.21, C43.22, C43.30, C43.31, C43.39, C43.4, C43.51, C43.52, C43.59, C43.60, C43.61, C43.62, C43.70, C43.71, C43.72, C43.8, C43.9, C45.0, C45.1, C45.2, C45.7, C45.9, C46.0, C46.1, C46.2, C46.3, C46.4, C46.50, C46.51, C46.52, C46.7, C46.9, C47.0, C47.10, C47.11, C47.12, C47.20, C47.21, C47.22, C47.3, C47.4, C47.5, C47.6, C47.8, C47.9, C48.0, C48.1, C48.2, C48.8, C49.0, C49.10, C49.11, C49.12, C49.20, C49.21, C49.22, C49.x (x=3, 4, 5, 6, 8, 9), C49.Ax (x=0, 1, 2,3 ,4, 5, 9), C4A.0, C4A.1x (x=0, 1, 2), C4A.2x (x=0, 1, 2),  C4A.3x (x=0, 1, 9), C4A.4, C4A.5x (x=1, 2, 9), C4A.6x (x=0, 1, 2), C4A.7x (x=0, 1, 2), C4A.8, C4A.9, C50.x11 (x=0, 1, 2, 3, 4, 5, 6, 8, 9), C50.x12 (x=0, 1, 2, 3, 4, 5, 6, 8, 9), C50.x19 (x=0, 1, 2, 3, 4, 5, 6, 8, 9), C50.x21 (x=0, 1, 2, 3, 4, 5, 6, 8, 9), C50.x22 (x=0, 1, 2, 3, 4, 5, 6, 8, 9), C50.x29 (x=0, 1, 2, 3, 4, 5, 6, 8, 9), C51.x (x=0, 1, 2, 8, 9), C52, C53.x (x=0, 1, 3, 8, 9), C54.x (x=2, 3, 8, 9), C55, C56.1, C56.2, C56.9, C57.x0 (x=0, 1, 2), C57.x1 (x=0, 1, 2), C57.02 (x=0, 1, 2), C57.3, C57.4, C57.7, C57.8, C57.9, C58, C60.x (x=0, 1, 2, 8, 9), C61, C62.0x (x=0, 1, 2), C62.1x (x=0, 1, 2), C62.9x (x=0, 1, 2), C63.0x (x=0, 1, 2), C63.1x (x=0, 1, 2), C63.2, C63.7, C63.8, C63.9, C64.1, C64.2, C64.9, C65.1, C65.2, C65.9, C66.1, C66.2, C66.9, C67.x (x=0 to 9), C68.x (x=0, 1, 8, 9), C69.x0 (x=0, 1, 2, 3, 4, 5, 6, 8, 9), C69.x1 (x=0, 1, 2, 3, 4, 5, 6, 8, 9), C69.x2 (x=0, 1, 2, 3, 4, 5, 6, 8, 9), C70.0, C70.1, C70.9, C71.x, C72.0, C72.1, C72.x0 (x=2 to 5), C72.x1 (x=2 to 4), C72.x2 (x=2 to 4), C72.59, C72.9, C73, C74.0x (x=0, 1, 2), C74.1x (x=0, 1, 2), C74.9x (x=0, 1, 2), C75.x (x=0, 1, 2, 3, 4, 5, 8, 9). C76.x (x=0 to 3), C76.4x (x=0, 1, 2), C76.5x (x=0, 1, 2, 8), C77.x (x=0, 1, 2, 3, 4, 5, 8, 9), C78.0x (x=0, 1, 2), C78.1, C78.2, C78.30, C78.39, C78.4, C78.5, C78.6, C78.7, C78.80, C78.89, C79.0x (x=0, 1, 2), C79.10, C79.11, C79.19, C79.2, C79.31, C79.32, C79.40, C79.49, C79.51, C79.52, C79.6x (x=0, 1, 2), C79.7x (0, 1, 2), C79.8x (x=1, 2, 9), C79.9, C7A.00, C7A.010, C7A.011, C7A.012, C7A.019, C7A.02x (x=0 to 6, and 9), C7A.09x (x=0 to 6 and 8), C7A.1, C7A.8, C7B.0x (x=0, 1, 2, 3, 4, 9), C7B.1, C7B.8, C81.0x, C81.1x, C81.2x, C81.3x, C81.4x, C81.7x, C81.9x, C82.0x, C82.1x, C82.2x, C82.3x, C82.4x, C82.5x, C82.6x, C82.8x, C82.9x, C83.0x, C83.1x, C83.3x, C83.5x, C83.7x, C83.8x, C83.9x, C84.0x, C84.1x, C84.4x, C84.6x, C84.7x, C84.9x, C84.Ax, C84.Zx, C85.1x, C85.2x, C85.8x, C85.9x, C86.x (x=0 to 6), C88.x (x=0, 2, 3, 4, 8, 9), C90.x0 (x=0 to 3), C90.x1 (x=0 to 3), C90.x2 (x=0 to 3), C91.x0 (x=A, Z, 0, 1, 3, 4, 5, 6, 9), C91.x1 (x=A, Z, 0, 1, 3, 4, 5, 6, 9), C92.x2 (x=A, Z, 0, 1, 3, 4, 5, 6, 9), C92.9x (x=0, 1, 2), C92.Ax (x=0, 1, 2), C92.Zx (x=0, 1, 2), C93.0x (x=0, 1, 2), C93.1x (x=0, 1, 2), C93.3x (x=0, 1, 2), C93.9x (x=0, 1, 2), C93.Zx (x=0, 1, 2), C94.0x (x=0, 1, 2), C94.2x (x=0, 1, 2), C94.3x (x=0, 1, 2), C94.4x (x=0, 1, 2), C94.6, C94.8x (x=0, 1, 2), C95.0x (x=0, 1, 2), C95.1x (x=0, 1, 2), C95.9x (x=0, 1, 2), C96.x (x=0, 2, 4, 5, 6, 9, A, Z) |
