# Supplementary material for: Using machine learning to predict risk of incident opioid use disorder among fee-for-service Medicare beneficiaries: A prognostic study
Source: PLoS One. 2020 Jul 17;15(7):e0235981. doi: 10.1371/journal.pone.0235981 (PMC7367453; doi:10.1371/journal.pone.0235981)
Supplement: S2 Table — (DOCX) [file pone.0235981.s005.docx]

**S2 Table. Diagnosis codes for identifying opioid use disorder and opioid overdose**

| **Conditions** | **ICD-9 codes** | **ICD-10 codes** |
| --- | --- | --- |
| Opioid overdose | 965.00, 965.01, 965.02, 965.09, E850.0, E850.1, E850.2, E935.0, E935.1, E935.2 | T40.0X1A, T40.0X2A, T40.0X3A, T40.0X4A, T40.1X1A, T40.1X2A, T40.1X3A, T40.1X4A, T40.2X1A. T40.2X2A, T40.2X3A, T40.2X4A, T40.3X1A, T40.3X2A, T40.3X3A, T40.3X4A, T40.4X1A, T40.4X2A, T40.4X3A, T40.4X4A, T40.601A, T40.602A, T40.603A, T40.604A, T40.691A, T40.692A, T40.693A, T40.694A |
| Incident opioid use disorder | 304.0X, 304.7X, 305.5X | F11.1X and F11.2X, excluding F11.11 (opioid-related disorders in remission) and F11.21 (opioid dependence in remission) |
