# Supplementary material for: Using machine learning to predict risk of incident opioid use disorder among fee-for-service Medicare beneficiaries: A prognostic study
Source: PLoS One. 2020 Jul 17;15(7):e0235981. doi: 10.1371/journal.pone.0235981 (PMC7367453; doi:10.1371/journal.pone.0235981)
Supplement: S4 Table — (DOCX) [file pone.0235981.s007.docx]

**S4 Table. Summary of predictor candidates (n=269) measured in 3-month windows for predicting incident opioid use disorder or opioid overdose ^a^**

| **Patterns of prescription opioid use^b^** | **Patterns of non-opioid prescription use** | **Beneficiaries sociodemographics** | **Health status factors** | **Opioid prescriber-level variables^d^** | **Regional-level factors^e^** |
| --- | --- | --- | --- | --- | --- |
| - Average opioid daily dose in MME^c^ - Cumulative MME - Cumulative duration for any opioids, SAO, and LAO - Duration of longest continuous use for any opioids, SAO, and LAO - No. fills of any opioids, SAO, and LAO - No. standardized 30-day prescriptions for any opioids, SAO, and LAO - Cumulative duration of 30-day use of any opioids, SAO, and LAO - No. fills by opioid ingredient and type (e.g., any fentanyl, SAO-type fentanyl, LAO-type fentanyl) - Type of opioids by Schedule and SAO/LAO (e.g., SAO, Schedule I only) - No. unique opioid prescribers - No. unique pharmacies - No. early refills for opioids - Cumulative overlapping days of early refills - Use of injectible opioids or antitussive opioids | - No. BZD fills - No. muscle relaxants fills - Cumulative overlapping days of concurrent opioid and BZD use - Cumulative overlapping days of concurrent opioid and muscle relaxants use - Cumulative overlapping days of concurrent opioid, BZD and muscle relaxants use - Cumulative duration of buprenorphine for opioid use disorder - Cumulative duration of naltrexone - No. gabapentinoid fills - Cumulative duration of gabapentinoid use - No. antidepressants fills - Cumulative duration of antidepressant use - No. average monthly non-opioid prescriptions - No. naltrexone fills | - Age - Sex - Race - State of residence - County of residence - Zip code of residence - Type of county of residence (metro vs. non-metro) - Disabled eligibility - Receipt of low-income subsidy | - No. outpatient visits - No. ED visits - No. inpatient visits - History of prescription opioid overdose - History of heroin overdose - Non-opioid drug use disorders - Other non-opioid SUD or alcohol use disorders - Alcohol use disorders - History of urine drug tests - History of SUD counseling - Adjustment disorders - Personality disorders - Psychoses - Delusional disorders - Schizophrenia - Mood disorders - Anxiety disorders - Alcohol-induced mental disorders - Drug-induced mental or sleep disorders - Other mental health disorders - Osteoarthritis - Rheumatoid arthritis - Back pain - Neck pain - Headache or migraine - Temporomandibular disorder pain - Abdominal pain or hernia - Chest pain - Kidney or gall bladder stones - Menstrual or genital reproductive pain - Fractures, concussion, injuries - Fibromyalgia - Internal orthopedic device implant/graft - Other pain conditions - Surgical procedures (e.g., ischemic heart diseases) - Diseases of musculoskeletal system and connective tissues - Neuropathies (excluding alcoholic, drug, and optic-related) - Ischemic heart disease - HIV/AIDS - Elixhauser index and individual categories | - Prescriber’s sex - Prescriber’s specialties - Average monthly opioid prescribing volume - Average monthly opioid prescribing dose in MME - Average monthly patients receiving opioids | - AHRF total health facilities variables - AHRF health professions variables - AHRF resource scarcity variables - AHRF health training programs variables - AHRF hospital expenditure, Medicare costs, VA expenditure - AHRF inpatient days/discharges variables - AHRF other health services utilization variables - AHRF census-based variables (e.g., medium household income, employment) - AHRF health insurance status variables - AHRF housing statistics - Area deprivation index - County-health ranking variables |

Abbreviations: AHRF: Area Health Resources Files; BZD: benzodiazepines; LAO: long-acting opioids; MME: morphine milligram equivalent; No: Number of; SAO: short-acting opioids; SUD: substance use disorders;
^a:^ Details for the operational definitions for each variable and corresponding diagnosis and procedure codes and National Drug Codes can be provided per request to the corresponding author.
^b:^ We used an “as-prescribed” approach that assumes patients taking all prescribed opioids on the schedule recommended by their clinicians. **(**Bohnert AS et al. JAMA. 2011;305(13):1315-21. doi: 10.1001/jama.2011.370.) Patients who received refills for the same drug at the same dose and schedule while still having opioid prescriptions within three days from a prior fill were assumed to have taken the medication from the prior fill before taking medication from the second fill. (Gellad WF et al. Am J Public Health. 2018;108(2):248-255. doi: 10.2105/AJPH.2017.304174.)

^c:^ We calculated morphine milligram equivalent (MME) for each opioid prescription, defined by the quantity dispensed multiplied by the strength in milligrams, multiplied by a conversion factor. **(**Bohnert AS et al. JAMA. 2011;305(13):1315-21. doi: 10.1001/jama.2011.370.) For each person, the average daily MME during the 3-month window was calculated by summing MMEs across all opioids and dividing by the number of days supplied.

^d:^ Prescribers were identified by their National Provider Identifiers. Primary opioid prescribers were defined as the prescribers who dominantly prescribed the most opioid prescriptions. If patients only had two opioid prescriptions, then the first prescriber was considered as the primary prescriber.

^e^: AHRF variables (<https://data.hrsa.gov/topics/health-workforce/ahrf>), area deprivation index (<https://www.hipxchange.org/ADI>), and county-health ranking variables (<http://www.countyhealthrankings.org/explore-health-rankings/use-data>) are publicly available and downloadable.
f: Methadone for opioid use disorder was identified using the procedure codes (H0020, J1230) and buprenorphine for opioid use disorder was identified from prescription sublingual buprenorphine or buprenorphine/naloxone using NDC codes.
