# Supplementary material for: Using machine learning to predict risk of incident opioid use disorder among fee-for-service Medicare beneficiaries: A prognostic study
Source: PLoS One. 2020 Jul 17;15(7):e0235981. doi: 10.1371/journal.pone.0235981 (PMC7367453; doi:10.1371/journal.pone.0235981)
Supplement: S5 Table — (DOCX) [file pone.0235981.s008.docx]

**S5 Table. Prediction performance measures for predicting incident opioid use disorder, across different machine learning methods with varying sensitivity and specificity.**

| **Methods** | **Score threshold (range 0-100)^a^** | **Predicted overdose (%)** | **Sensitivity (%)** | **Specificity (%)** | **PPV (%)** | **NPV (%)** | **F1 score (%)** | **PLR** | **NNE** |
| --- | --- | --- | --- | --- | --- | --- | --- | --- | --- |
| **Elastic Net** |  |  |  |  |  |  |  |  |  |
| **Sensitivity** |  |  |  |  |  |  |  |  |  |
| **100%** | 6.48 | 99.14 | 100.00 | 0.86 | 0.15 | 100.0 | 0.0029 | 1.01 | 690 |
| 99% | 10.45 | 86.34 | 98.98 | 13.68 | 0.16 | 99.99 | 0.0033 | 1.15 | 607 |
| 98% | 12.84 | 72.86 | 98.02 | 27.18 | 0.19 | 99.99 | 0.0039 | 1.35 | 517 |
| 97% | 15.91 | 59.30 | 97.00 | 40.75 | 0.24 | 99.99 | 0.0047 | 1.64 | 425 |
| 96% | 17.81 | 53.89 | 95.99 | 46.17 | 0.26 | 99.99 | 0.0051 | 1.78 | 390 |
| 95% | 20.16 | 49.02 | 95.02 | 51.05 | 0.28 | 99.99 | 0.0056 | 1.94 | 359 |
| 94% | 22.93 | 44.58 | 94.01 | 55.49 | 0.30 | 99.98 | 0.0060 | 2.11 | 330 |
| 93% | 25.68 | 40.67 | 92.99 | 59.40 | 0.33 | 99.98 | 0.0066 | 2.29 | 304 |
| 92% | 27.77 | 37.85 | 91.97 | 62.23 | 0.35 | 99.98 | 0.0070 | 2.43 | 286 |
| 91% | 29.26 | 35.84 | 91.01 | 64.24 | 0.37 | 99.98 | 0.0073 | 2.54 | 274 |
| 90% | 31.08 | 33.50 | 89.99 | 66.59 | 0.39 | 99.98 | 0.0077 | 2.69 | 259 |
| **Optimized threshold^b^** | 42.25 | 21.57 | 81.49 | 78.51 | 0.54 | 99.97 | 0.0108 | 3.79 | 184 |
| **Specificity** |  |  |  |  |  |  |  |  |  |
| 90% | 60.28 | 10.08 | 65.86 | 90.00 | 0.94 | 99.95 | 0.0185 | 6.59 | 106 |
| 91% | 62.74 | 9.08 | 63.35 | 91.00 | 1.00 | 99.94 | 0.0197 | 7.04 | 100 |
| 92% | 65.52 | 8.08 | 60.67 | 92.00 | 1.08 | 99.94 | 0.0212 | 7.58 | 93 |
| 93% | 68.70 | 7.08 | 57.89 | 93.00 | 1.18 | 99.93 | 0.0231 | 8.27 | 85 |
| 94% | 72.27 | 6.07 | 54.68 | 94.00 | 1.29 | 99.93 | 0.0253 | 9.11 | 77 |
| 95% | 76.15 | 5.07 | 51.42 | 95.00 | 1.46 | 99.93 | 0.0284 | 10.28 | 69 |
| 96% | 80.41 | 4.06 | 47.35 | 96.00 | 1.68 | 99.92 | 0.0324 | 11.84 | 60 |
| 97% | 84.90 | 3.06 | 41.73 | 97.00 | 1.96 | 99.91 | 0.0375 | 13.91 | 51 |
| 98% | 89.61 | 2.05 | 34.24 | 98.00 | 2.41 | 99.90 | 0.0449 | 17.12 | 42 |
| 99% | 94.50 | 1.03 | 22.69 | 99.00 | 3.17 | 99.89 | 0.0556 | 22.73 | 32 |
| **100%** | 99.84 | 0.00 | 0.00 | 100.00 | 0.00 | 99.86 | nan | 0.00 | inf |
| **Maximized PPV** | 99.82 | 0.00 | 0.05 | 100.00 | 33.33 | 99.86 | 0.0011 | 347.30 | 3 |
| **GBM** |  |  |  |  |  |  |  |  |  |
| **Sensitivity** |  |  |  |  |  |  |  |  |  |
| **100%** | 5.36 | 99.36 | 100.00 | 0.64 | 0.14 | 100.00 | 0.0029 | 1.01 | 691 |
| 99% | 9.47 | 79.52 | 98.98 | 20.51 | 0.18 | 99.99 | 0.0036 | 1.25 | 559 |
| 98% | 11.26 | 68.93 | 98.02 | 31.11 | 0.20 | 99.99 | 0.0041 | 1.42 | 489 |
| 97% | 12.67 | 62.21 | 97.00 | 37.84 | 0.22 | 99.99 | 0.0045 | 1.56 | 446 |
| 96% | 14.31 | 56.00 | 95.99 | 44.06 | 0.25 | 99.99 | 0.0049 | 1.72 | 406 |
| 95% | 16.19 | 50.31 | 95.02 | 49.76 | 0.27 | 99.99 | 0.0054 | 1.89 | 368 |
| 94% | 17.52 | 47.12 | 94.01 | 52.94 | 0.29 | 99.98 | 0.0057 | 2.00 | 349 |
| 93% | 19.60 | 42.96 | 92.99 | 57.11 | 0.31 | 99.98 | 0.0062 | 2.17 | 321 |
| 92% | 21.88 | 39.27 | 91.97 | 60.81 | 0.34 | 99.98 | 0.0067 | 2.35 | 297 |
| 91% | 24.13 | 36.29 | 91.01 | 63.79 | 0.36 | 99.98 | 0.0072 | 2.51 | 277 |
| 90% | 26.81 | 33.41 | 89.99 | 66.67 | 0.39 | 99.98 | 0.0077 | 2.70 | 258 |
| **Optimized threshold^a^** | 46.01 | 19.67 | 80.42 | 80.42 | 0.59 | 99.96 | 0.0117 | 4.11 | 170 |
| **Specificity** |  |  |  |  |  |  |  |  |  |
| 90% | 58.68 | 10.08 | 66.29 | 90.00 | 0.95 | 99.95 | 0.0186 | 6.63 | 106 |
| 91% | 60.43 | 9.08 | 64.69 | 91.00 | 1.02 | 99.94 | 0.0202 | 7.19 | 98 |
| 92% | 62.52 | 8.08 | 62.12 | 92.00 | 1.11 | 99.94 | 0.0217 | 7.76 | 90 |
| 93% | 65.02 | 7.08 | 59.39 | 93.00 | 1.21 | 99.94 | 0.0237 | 8.48 | 83 |
| 94% | 67.88 | 6.08 | 56.34 | 94.00 | 1.33 | 99.93 | 0.0260 | 9.39 | 75 |
| 95% | 71.16 | 5.07 | 52.54 | 95.00 | 1.49 | 99.93 | 0.0290 | 10.51 | 67 |
| 96% | 75.01 | 4.06 | 48.21 | 96.00 | 1.71 | 99.92 | 0.0329 | 12.05 | 59 |
| 97% | 79.48 | 3.06 | 41.73 | 97.00 | 1.96 | 99.91 | 0.0375 | 13.91 | 51 |
| 98% | 84.47 | 2.05 | 33.98 | 98.00 | 2.39 | 99.90 | 0.0446 | 16.99 | 42 |
| 99% | 89.35 | 1.03 | 22.31 | 99.00 | 3.12 | 99.89 | 0.0547 | 22.34 | 32 |
| **100%** | 95.65 | 0.00 | 0.00 | 100.00 | 0.00 | 99.86 | nan | 0.00 | inf |
| **Maximized PPV** | 95.48 | 0.00 | 0.11 | 100.00 | 16.67 | 99.86 | 0.0021 | 138.92 | 6 |

**S5 Table (continued).**

| **Methods** | **Score threshold (range 0-100)^a^** | **Predicted OUD (%)** | **Sensitivity (%)** | **Specificity (%)** | **PPV (%)** | **NPV (%)** | **F1 score (%)** | **PLR** | **NNE** |
| --- | --- | --- | --- | --- | --- | --- | --- | --- | --- |
| **RF** |  |  |  |  |  |  |  |  |  |
| **Sensitivity** |  |  |  |  |  |  |  |  |  |
| **100%** | 11.94 | 99.88 | 100.00 | 0.12 | 0.14 | 100.00 | 0.0029 | 1.00 | 695 |
| 99% | 14.52 | 84.23 | 98.98 | 15.79 | 0.17 | 99.99 | 0.0034 | 1.18 | 592 |
| 98% | 16.19 | 73.87 | 98.02 | 26.16 | 0.19 | 99.99 | 0.0038 | 1.33 | 524 |
| 97% | 18.36 | 65.25 | 97.00 | 34.80 | 0.21 | 99.99 | 0.0043 | 1.49 | 468 |
| 96% | 20.70 | 58.91 | 95.99 | 41.15 | 0.23 | 99.99 | 0.0047 | 1.63 | 427 |
| 95% | 23.71 | 50.51 | 95.02 | 49.56 | 0.27 | 99.99 | 0.0054 | 1.88 | 370 |
| 94% | 25.49 | 45.93 | 94.01 | 54.14 | 0.29 | 99.98 | 0.0059 | 2.05 | 340 |
| 93% | 26.75 | 43.40 | 92.99 | 56.67 | 0.31 | 99.98 | 0.0061 | 2.15 | 325 |
| 92% | 28.35 | 40.53 | 91.97 | 59.54 | 0.33 | 99.98 | 0.0065 | 2.27 | 307 |
| 91% | 30.37 | 37.61 | 91.01 | 62.47 | 0.35 | 99.98 | 0.0069 | 2.43 | 287 |
| 90% | 32.02 | 35.78 | 89.99 | 64.30 | 0.36 | 99.98 | 0.0072 | 2.52 | 277 |
| **Optimized threshold^a^** | 46.16 | 21.28 | 80.26 | 78.81 | 0.54 | 99.96 | 0.0108 | 3.79 | 184 |
| **Specificity** |  |  |  |  |  |  |  |  |  |
| 90% | 55.31 | 10.08 | 64.58 | 90.00 | 0.92 | 99.94 | 0.0182 | 6.46 | 109 |
| 91% | 56.90 | 9.08 | 62.01 | 91.00 | 0.98 | 99.94 | 0.0193 | 6.89 | 102 |
| 92% | 58.82 | 8.07 | 59.50 | 92.00 | 1.06 | 99.94 | 0.0208 | 7.44 | 94 |
| 93% | 61.23 | 7.07 | 57.09 | 93.00 | 1.16 | 99.93 | 0.0228 | 8.16 | 86 |
| 94% | 64.22 | 6.07 | 54.15 | 94.00 | 1.28 | 99.93 | 0.0251 | 9.02 | 78 |
| 95% | 67.89 | 5.06 | 50.67 | 95.00 | 1.44 | 99.93 | 0.0280 | 10.14 | 70 |
| 96% | 72.16 | 4.07 | 46.76 | 95.99 | 1.65 | 99.92 | 0.0319 | 11.66 | 61 |
| 97% | 76.95 | 3.05 | 41.36 | 97.01 | 1.95 | 99.91 | 0.0373 | 13.82 | 51 |
| 98% | 81.99 | 2.04 | 33.81 | 98.01 | 2.39 | 99.90 | 0.0446 | 16.99 | 42 |
| 99% | 86.93 | 1.04 | 21.88 | 98.99 | 3.04 | 99.89 | 0.0534 | 21.77 | 33 |
| **100%** | 95.01 | 0.00 | 0.00 | 100.00 | 0.00 | 99.86 | nan | 0.00 | inf |
| **Maximized PPV** | 94.23 | 0.01 | 0.37 | 99.99 | 6.73 | 99.86 | 0.0071 | 50.13 | 15 |
| **DNN** |  |  |  |  |  |  |  |  |  |
| **Sensitivity** |  |  |  |  |  |  |  |  |  |
| **100%** | 0.44 | 99.75 | 100.00 | 0.25 | 0.14 | 100.00 | 0.0029 | 1.00 | 694 |
| 99% | 2.25 | 82.73 | 98.98 | 17.30 | 0.17 | 99.99 | 0.0034 | 1.20 | 581 |
| 98% | 3.66 | 70.34 | 98.02 | 29.70 | 0.20 | 99.99 | 0.0040 | 1.39 | 499 |
| 97% | 5.65 | 59.02 | 97.00 | 41.03 | 0.24 | 99.99 | 0.0047 | 1.65 | 423 |
| 96% | 7.48 | 52.38 | 95.99 | 47.68 | 0.26 | 99.99 | 0.0053 | 1.83 | 380 |
| 95% | 8.87 | 48.63 | 95.02 | 51.44 | 0.28 | 99.99 | 0.0056 | 1.96 | 356 |
| 94% | 10.33 | 45.51 | 94.01 | 54.56 | 0.30 | 99.98 | 0.0059 | 2.07 | 337 |
| 93% | 11.47 | 43.40 | 92.99 | 56.67 | 0.31 | 99.98 | 0.0061 | 2.15 | 325 |
| 92% | 13.68 | 39.98 | 91.97 | 60.09 | 0.33 | 99.98 | 0.0066 | 2.30 | 302 |
| 91% | 17.17 | 35.77 | 91.01 | 64.30 | 0.37 | 99.98 | 0.0073 | 2.55 | 273 |
| 90% | 18.55 | 34.34 | 89.99 | 65.74 | 0.38 | 99.98 | 0.0075 | 2.63 | 265 |
| **Optimized threshold^a^** | 40.10 | 19.22 | 79.13 | 80.87 | 0.59 | 99.96 | 0.0118 | 4.14 | 169 |
| **Specificity** |  |  |  |  |  |  |  |  |  |
| 90% | 59.47 | 10.08 | 65.38 | 90.00 | 0.93 | 99.94 | 0.0184 | 6.54 | 107 |
| 91% | 62.18 | 9.08 | 63.72 | 91.00 | 1.01 | 99.94 | 0.0199 | 7.08 | 99 |
| 92% | 65.08 | 8.08 | 61.74 | 92.00 | 1.10 | 99.94 | 0.0216 | 7.72 | 91 |
| 93% | 68.24 | 7.08 | 59.28 | 93.00 | 1.20 | 99.94 | 0.0236 | 8.47 | 83 |
| 94% | 71.71 | 6.07 | 56.45 | 94.00 | 1.34 | 99.93 | 0.0261 | 9.41 | 75 |
| 95% | 75.56 | 5.07 | 52.70 | 95.00 | 1.49 | 99.93 | 0.0291 | 10.54 | 67 |
| 96% | 79.61 | 4.06 | 47.78 | 96.00 | 1.69 | 99.92 | 0.0326 | 11.94 | 59 |
| 97% | 84.01 | 3.06 | 43.07 | 97.00 | 2.03 | 99.92 | 0.0387 | 14.36 | 49 |
| 98% | 88.30 | 2.05 | 36.01 | 98.00 | 2.52 | 99.91 | 0.0472 | 17.99 | 40 |
| 99% | 92.33 | 1.03 | 24.61 | 99.00 | 3.42 | 99.89 | 0.0601 | 24.59 | 29 |
| **100%** | 99.27 | 0.00 | 0.00 | 100.00 | 0.00 | 99.86 | nan | 0.00 | inf |
| **Maximized PPV** | 99.26 | 0.00 | 0.05 | 100.00 | 50.00 | 99.86 | 0.0011 | 694.59 | 2 |

Abbreviations: DNN: deep neural network; GBM: gradient boosting machine; INF: infinity; N/A: not able to calculated; NNE: number needed to evaluate; NPV: negative predictive values; PLR: positive likelihood ratio; PPV: positive predictive values; RF: random forest.
^a^: Scores were calculated by predicted probability multiplied by 100. Score threshold refers to the score used to classify or predict individuals with OUD (i.e., ≥ the threshold) vs. non-OUD (i.e., <threshold)
^b^: Optimized threshold was calculated by the Youden Index to achieve balanced sensitivity and specificity.
