# Supplementary material for: Using machine learning to predict risk of incident opioid use disorder among fee-for-service Medicare beneficiaries: A prognostic study
Source: PLoS One. 2020 Jul 17;15(7):e0235981. doi: 10.1371/journal.pone.0235981 (PMC7367453; doi:10.1371/journal.pone.0235981)
Supplement: S6 Table — (DOCX) [file pone.0235981.s009.docx]

**S6 Table. Prediction performance measures for predicting incident opioid use disorder or opioid overdose, across different machine learning methods with varying sensitivity and specificity.**

| **Methods** | **Score threshold (range 0-100)^a^** | **Predicted overdose (%)** | **Sensitivity (%)** | **Specificity (%)** | **PPV (%)** | **NPV (%)** | **F1 score (%)** | **PLR** | **NNE** |
| --- | --- | --- | --- | --- | --- | --- | --- | --- | --- |
| **DNN** |  |  |  |  |  |  |  |  |  |
| **Sensitivity** |  |  |  |  |  |  |  |  |  |
| **100%** | 8.37 | 94.22 | 100.00 | 5.79 | 0.19 | 100.00 | 0.0038 | 1.06 | 526 |
| 99% | 14.88 | 73.18 | 99.03 | 26.86 | 0.24 | 99.99 | 0.0048 | 1.35 | 413 |
| 98% | 19.60 | 61.37 | 98.06 | 38.70 | 0.29 | 99.99 | 0.0057 | 1.60 | 349 |
| 97% | 23.04 | 54.96 | 97.09 | 45.11 | 0.32 | 99.99 | 0.0063 | 1.77 | 316 |
| 96% | 26.31 | 49.99 | 96.12 | 50.09 | 0.34 | 99.99 | 0.0069 | 1.93 | 290 |
| 95% | 28.07 | 47.68 | 95.15 | 52.41 | 0.36 | 99.98 | 0.0071 | 2.00 | 280 |
| 94% | 30.12 | 44.99 | 94.17 | 55.10 | 0.38 | 99.98 | 0.0075 | 2.10 | 267 |
| 93% | 32.65 | 41.88 | 93.20 | 58.21 | 0.40 | 99.98 | 0.0079 | 2.23 | 251 |
| 92% | 35.00 | 39.05 | 92.23 | 61.04 | 0.42 | 99.98 | 0.0084 | 2.37 | 236 |
| 91% | 38.82 | 34.74 | 90.78 | 65.37 | 0.47 | 99.97 | 0.0093 | 2.62 | 214 |
| 90% | 40.29 | 33.15 | 89.81 | 66.95 | 0.49 | 99.97 | 0.0097 | 2.72 | 206 |
| **Optimized threshold^b^** | 55.17 | 19.26 | 81.07 | 80.85 | 0.75 | 99.96 | 0.0149 | 4.23 | 133 |
| **Specificity** |  |  |  |  |  |  |  |  |  |
| 90% | 70.02 | 10.16 | 67.48 | 89.94 | 1.19 | 99.94 | 0.0234 | 6.71 | 84 |
| 91% | 72.13 | 9.16 | 66.02 | 90.94 | 1.29 | 99.93 | 0.0253 | 7.29 | 77 |
| 92% | 74.46 | 8.14 | 62.14 | 91.96 | 1.37 | 99.93 | 0.0268 | 7.73 | 73 |
| 93% | 76.56 | 7.25 | 58.74 | 92.85 | 1.45 | 99.92 | 0.0283 | 8.21 | 69 |
| 94% | 79.34 | 6.14 | 54.85 | 93.95 | 1.60 | 99.91 | 0.0311 | 9.07 | 62 |
| 95% | 82.04 | 5.09 | 50.00 | 94.99 | 1.76 | 99.91 | 0.0340 | 9.97 | 57 |
| 96% | 84.72 | 4.08 | 45.63 | 95.99 | 2.00 | 99.90 | 0.0384 | 11.39 | 50 |
| 97% | 87.48 | 3.11 | 40.78 | 96.96 | 2.35 | 99.89 | 0.0444 | 13.40 | 43 |
| 98% | 90.70 | 2.05 | 33.01 | 98.00 | 2.88 | 99.88 | 0.0530 | 16.53 | 35 |
| 99% | 93.77 | 1.03 | 20.87 | 99.00 | 3.63 | 99.86 | 0.0618 | 20.96 | 28 |
| **100%** | 99.19 | 0.00 | 0.00 | 100.00 | 0.00 | 99.82 | nan | 0.00 | inf |
| **Maximized PPV** | 97.10 | 0.16 | 8.74 | 99.86 | 10.00 | 99.84 | 0.0933 | 61.91 | 10 |
| **GBM** |  |  |  |  |  |  |  |  |  |
| **Sensitivity** |  |  |  |  |  |  |  |  |  |
| **100%** | 8.85 | 97.58 | 100.00 | 2.42 | 0.18 | 100.00 | 0.0037 | 1.02 | 545 |
| 99% | 16.94 | 66.45 | 99.03 | 33.61 | 0.27 | 99.99 | 0.0053 | 1.49 | 375 |
| 98% | 19.47 | 59.54 | 98.06 | 40.53 | 0.30 | 99.99 | 0.0059 | 1.65 | 339 |
| 97% | 21.55 | 54.99 | 97.09 | 45.09 | 0.32 | 99.99 | 0.0063 | 1.77 | 316 |
| 96% | 22.61 | 52.98 | 96.12 | 47.10 | 0.33 | 99.99 | 0.0065 | 1.82 | 308 |
| 95% | 25.26 | 48.55 | 95.15 | 51.54 | 0.35 | 99.98 | 0.0070 | 1.96 | 285 |
| 94% | 28.16 | 44.50 | 94.17 | 55.59 | 0.38 | 99.98 | 0.0076 | 2.12 | 264 |
| 93% | 29.56 | 42.65 | 93.20 | 57.44 | 0.39 | 99.98 | 0.0078 | 2.19 | 255 |
| 92% | 33.81 | 37.89 | 92.23 | 62.20 | 0.44 | 99.98 | 0.0087 | 2.44 | 229 |
| 91% | 40.43 | 31.74 | 90.78 | 68.37 | 0.51 | 99.98 | 0.0102 | 2.87 | 195 |
| 90% | 44.16 | 28.41 | 89.81 | 71.70 | 0.57 | 99.97 | 0.0113 | 3.17 | 177 |
| **Optimized threshold^a^** | 52.63 | 20.80 | 83.50 | 79.31 | 0.72 | 99.96 | 0.0143 | 4.04 | 139 |
| **Specificity** |  |  |  |  |  |  |  |  |  |
| 90% | 67.29 | 10.20 | 66.99 | 89.90 | 1.18 | 99.93 | 0.0231 | 6.63 | 85 |
| 91% | 69.28 | 9.12 | 65.53 | 90.99 | 1.29 | 99.93 | 0.0253 | 7.27 | 78 |
| 92% | 70.89 | 8.24 | 61.65 | 91.85 | 1.34 | 99.93 | 0.0262 | 7.57 | 75 |
| 93% | 73.20 | 7.08 | 59.22 | 93.02 | 1.50 | 99.92 | 0.0292 | 8.48 | 67 |
| 94% | 75.22 | 6.17 | 55.34 | 93.92 | 1.61 | 99.91 | 0.0312 | 9.10 | 62 |
| 95% | 77.69 | 5.11 | 51.46 | 94.97 | 1.80 | 99.91 | 0.0349 | 10.24 | 55 |
| 96% | 80.62 | 4.05 | 47.09 | 96.02 | 2.08 | 99.90 | 0.0399 | 11.84 | 48 |
| 97% | 83.66 | 3.03 | 40.78 | 97.03 | 2.41 | 99.89 | 0.0455 | 13.75 | 42 |
| 98% | 86.95 | 2.07 | 31.55 | 97.99 | 2.74 | 99.87 | 0.0504 | 15.68 | 37 |
| 99% | 90.74 | 1.03 | 18.45 | 99.00 | 3.19 | 99.85 | 0.0544 | 18.38 | 31 |
| **100%** | 95.80 | 0.00 | 0.00 | 100.00 | 0.00 | 99.82 | nan | 0.00 | inf |
| **Maximized PPV** | 95.43 | 0.01 | 0.97 | 99.99 | 18.18 | 99.82 | 0.0184 | 123.83 | 6 |

**S6 Table (continued).**

| **Methods** | **Score threshold (range 0-100)^a^** | **Predicted OUD (%)** | **Sensitivity (%)** | **Specificity (%)** | **PPV (%)** | **NPV (%)** | **F1 score (%)** | **PLR** | **NNE** |
| --- | --- | --- | --- | --- | --- | --- | --- | --- | --- |
| **Elastic Net** |  |  |  |  |  |  |  |  |  |
| **Sensitivity** |  |  |  |  |  |  |  |  |  |
| **100%** | 23.30 | 85.81 | 100.00 | 14.22 | 0.21 | 100.00 | 0.0042 | 1.17 | 479 |
| 99% | 26.05 | 71.70 | 99.03 | 28.35 | 0.25 | 99.99 | 0.0049 | 1.38 | 404 |
| 98% | 26.99 | 68.64 | 98.06 | 31.41 | 0.26 | 99.99 | 0.0051 | 1.43 | 391 |
| 97% | 29.85 | 61.84 | 97.09 | 38.22 | 0.28 | 99.99 | 0.0056 | 1.57 | 356 |
| 96% | 32.73 | 56.46 | 96.12 | 43.61 | 0.30 | 99.98 | 0.0061 | 1.70 | 328 |
| 95% | 34.74 | 54.01 | 95.15 | 46.07 | 0.32 | 99.98 | 0.0063 | 1.76 | 317 |
| 94% | 35.42 | 53.42 | 94.17 | 46.66 | 0.32 | 99.98 | 0.0063 | 1.77 | 317 |
| 93% | 37.39 | 51.97 | 93.20 | 48.11 | 0.32 | 99.97 | 0.0064 | 1.80 | 311 |
| 92% | 40.97 | 49.22 | 92.23 | 50.86 | 0.34 | 99.97 | 0.0067 | 1.88 | 298 |
| 91% | 43.47 | 46.05 | 90.78 | 54.03 | 0.35 | 99.97 | 0.0070 | 1.97 | 283 |
| 90% | 44.57 | 44.32 | 89.81 | 55.77 | 0.36 | 99.97 | 0.0072 | 2.03 | 275 |
| **Optimized threshold^a^** | 60.37 | 20.60 | 77.67 | 79.50 | 0.68 | 99.95 | 0.0134 | 3.79 | 148 |
| **Specificity** |  |  |  |  |  |  |  |  |  |
| 90% | 73.37 | 10.06 | 58.74 | 90.03 | 1.05 | 99.92 | 0.0206 | 5.89 | 96 |
| 91% | 75.44 | 9.09 | 56.31 | 90.99 | 1.11 | 99.91 | 0.0218 | 6.25 | 90 |
| 92% | 77.10 | 8.12 | 53.40 | 91.96 | 1.18 | 99.91 | 0.0230 | 6.64 | 85 |
| 93% | 78.59 | 7.08 | 52.43 | 93.00 | 1.33 | 99.91 | 0.0259 | 7.49 | 75 |
| 94% | 80.24 | 6.03 | 49.51 | 94.05 | 1.47 | 99.90 | 0.0286 | 8.32 | 68 |
| 95% | 81.85 | 5.07 | 45.15 | 95.00 | 1.59 | 99.90 | 0.0308 | 9.03 | 63 |
| 96% | 83.91 | 4.04 | 40.78 | 96.02 | 1.81 | 99.89 | 0.0346 | 10.25 | 55 |
| 97% | 86.21 | 3.03 | 33.50 | 97.02 | 1.98 | 99.88 | 0.0374 | 11.25 | 51 |
| 98% | 89.31 | 1.99 | 26.70 | 98.05 | 2.40 | 99.87 | 0.0440 | 13.69 | 42 |
| 99% | 93.07 | 1.00 | 19.42 | 99.04 | 3.49 | 99.85 | 0.0592 | 20.15 | 29 |
| **100%** | 97.80 | 0.00 | 0.00 | 100.00 | 0.00 | 99.82 | nan | 0.00 | inf |
| **Maximized PPV** | 97.63 | 0.00 | 0.49 | 100.00 | 50.00 | 99.82 | 0.0096 | 557.23 | 2 |
| **RF** |  |  |  |  |  |  |  |  |  |
| **Sensitivity** |  |  |  |  |  |  |  |  |  |
| **100%** | 20.24 | 90.71 | 100.00 | 9.31 | 0.20 | 100.00 | 0.0039 | 1.10 | 506 |
| 99% | 23.72 | 78.38 | 99.03 | 21.66 | 0.23 | 99.99 | 0.0045 | 1.26 | 442 |
| 98% | 27.32 | 69.49 | 98.06 | 30.56 | 0.25 | 99.99 | 0.0050 | 1.41 | 396 |
| 97% | 31.79 | 56.88 | 97.09 | 43.19 | 0.31 | 99.99 | 0.0061 | 1.71 | 327 |
| 96% | 33.32 | 53.55 | 96.12 | 46.52 | 0.32 | 99.99 | 0.0064 | 1.80 | 311 |
| 95% | 35.27 | 49.57 | 95.15 | 50.51 | 0.34 | 99.98 | 0.0069 | 1.92 | 291 |
| 94% | 37.77 | 45.29 | 94.17 | 54.80 | 0.37 | 99.98 | 0.0074 | 2.08 | 268 |
| 93% | 38.15 | 44.71 | 93.20 | 55.38 | 0.37 | 99.98 | 0.0074 | 2.09 | 268 |
| 92% | 38.28 | 44.51 | 92.23 | 55.57 | 0.37 | 99.97 | 0.0074 | 2.08 | 269 |
| 91% | 39.16 | 43.20 | 90.78 | 56.89 | 0.38 | 99.97 | 0.0075 | 2.11 | 266 |
| 90% | 40.55 | 41.18 | 89.81 | 58.91 | 0.39 | 99.97 | 0.0078 | 2.19 | 256 |
| **Optimized threshold^a^** | 56.47 | 18.25 | 76.21 | 81.85 | 0.75 | 99.95 | 0.0148 | 4.20 | 134 |
| **Specificity** |  |  |  |  |  |  |  |  |  |
| 90% | 64.66 | 10.26 | 63.59 | 89.84 | 1.11 | 99.93 | 0.0218 | 6.26 | 90 |
| 91% | 66.42 | 9.13 | 60.19 | 90.96 | 1.18 | 99.92 | 0.0232 | 6.66 | 85 |
| 92% | 68.27 | 8.09 | 58.74 | 92.00 | 1.30 | 99.92 | 0.0255 | 7.35 | 77 |
| 93% | 70.12 | 7.14 | 55.83 | 92.94 | 1.40 | 99.91 | 0.0273 | 7.91 | 71 |
| 94% | 72.69 | 5.99 | 50.49 | 94.09 | 1.51 | 99.91 | 0.0293 | 8.55 | 66 |
| 95% | 74.95 | 5.08 | 47.57 | 95.00 | 1.68 | 99.90 | 0.0324 | 9.51 | 60 |
| 96% | 77.85 | 3.98 | 44.66 | 96.09 | 2.01 | 99.90 | 0.0384 | 11.42 | 50 |
| 97% | 80.55 | 3.06 | 37.38 | 97.00 | 2.19 | 99.88 | 0.0413 | 12.47 | 46 |
| 98% | 83.68 | 2.08 | 28.64 | 97.97 | 2.47 | 99.87 | 0.0455 | 14.11 | 40 |
| 99% | 87.37 | 1.07 | 18.45 | 98.96 | 3.09 | 99.85 | 0.0530 | 17.79 | 32 |
| **100%** | 94.72 | 0.00 | 0.00 | 100.00 | 0.00 | 99.82 | nan | 0.00 | inf |
| **Maximized PPV** | 92.37 | 0.13 | 4.37 | 99.88 | 6.21 | 99.83 | 0.0513 | 36.88 | 16 |

Abbreviations: DNN: deep neural network; GBM: gradient boosting machine; INF: infinity; N/A: not able to calculated; NNE: number needed to evaluate; NPV: negative predictive values; PLR: positive likelihood ratio; PPV: positive predictive values; RF: random forest.
^a^: Scores were calculated by predicted probability multiplied by 100. Score threshold refers to the score used to classify or predict individuals with OUD (i.e., ≥ the threshold) vs. non-OUD (i.e., <threshold)
^b^: Optimized threshold was calculated by the Youden Index to achieve balanced sensitivity and specificity.
