# Supplementary material for: Using machine learning to predict risk of incident opioid use disorder among fee-for-service Medicare beneficiaries: A prognostic study
Source: PLoS One. 2020 Jul 17;15(7):e0235981. doi: 10.1371/journal.pone.0235981 (PMC7367453; doi:10.1371/journal.pone.0235981)
Supplement: S7 Table — (DOCX) [file pone.0235981.s010.docx]

**S7 Table. Comparison of prediction performance using any Centers for Medicare & Medicaid Services (CMS) high-risk opioid use measures vs. Deep Neural Network (DNN) and Gradient Boosting Machine (GBM) in the Validation sample (n=114,253) over a 12-month period^a^**

|  | **Any CMS measures^b^** | | **High risk defined as using different DNN’s thresholds ^c^** | | | **High risk defined as using different GBM’s thresholds ^c^** | | |
| --- | --- | --- | --- | --- | --- | --- | --- | --- |
| **Risk subgroups (n, % of the cohort)** | **Low risk  (n=110,171, 96.4%)** | **High risk  (n=4,082, 3.6%)** | **Top 1 percentile  (n=2,213, 1.9%)** | **Top 5^th^ percentile  (n=11,093, 9.7%)** | **Top 10^th^ percentile (n=20,654,  18.1%)** | **Top 1 percentile  (n=2,115, 1.9%)** | **Top 5^th^ percentile  (n=11,211 9.8%)** | **Top 10^th^ percentile (n=22,232,  19.5%)** |
| Number of actual OUD (% of each subgroup) | 412 (0.37) | 155 (3.80) | 187 (8.5) | 378 (3.41) | 452 (2.19) | 173 (8.18) | 373 (3.33) | 468 (2.11) |
| Number of actual non-OUD (% of each subgroup) | 109,759 (99.6) | 3,927 (96.2) | 2,026 (91.55) | 10,715 (96.59) | 20,202 (97.81) | 1,942 (91.82) | 10,838 (96.67) | 21,764 (97.89) |
| NNE | 270 | 26 | 11 | 29 | 45 | 12 | 30 | 47 |
| Overall number misclassified (% of overall cohort)^b^ | 412 (0.36) | 3,927 (3.4) | 2,026 (1.8) | 10,715 (9.4) | 20,202 (17.7) | 1,942 (1.7) | 10,838 (9.5) | 21,764 (19.1) |
| % of all OUD over 12 months (n=567) captured | 72.7 | 27.3 | 33.0 | 66.7 | 79.7 | 30.5 | 65.8 | 82.5 |

Abbreviations: N/A: not able to calculate; NNE: number needed to evaluate; OUD: opioid use disorder
^a^: The CMS measures were based on a 12-month period rather than 3 months. To compare CMS measures, beneficiaries were required to have at least a 12-month period of follow up, and thus the sample size was smaller than in the main analysis. If classifying beneficiaries with any of CMS high-risk opioid use measures as OUD, the remaining will be considered as non-OUD.
^b^: The 2019 CMS’ opioid safety measures, which are meant to identify high-risk individuals or utilization behavior.( Centers for Medicare and Medicaid Services [cited 2018 Nov 6]. Available from: <https://www.cms.gov/Medicare/Health-Plans/MedicareAdvtgSpecRateStats/Downloads/Announcement2019.pdf>.) These measures include 3 metrics (1) high-dose use, defined as >120 MME for ≥90 continuous days, (2) ≥4 opioid prescribers and ≥4 pharmacies, (3) concurrent opioid and benzodiazepine use ≥30 days.
^c^: For DNN, GBM, and RF, we presented high risk groups using different cutoff thresholds of prediction probability from the training sample: individuals with (1) predicted probability in the top 1 percentile (DNN=0.93, and GBM=0.90); (2) predicted probability in the top 2^nd^-5^th^ percentile (DNN=0.76, and GBM=0.72); and (3) predicted probability in the top 6^th^-10^th^ percentile (DNN=0.6, and GBM=0.59). If classifying beneficiaries in the high-risk group of OUD, the remaining will be consider as non-OUD.
