# Supplementary figures and images for: Using machine learning to predict risk of incident opioid use disorder among fee-for-service Medicare beneficiaries: A prognostic study
Source: PLoS One. 2020 Jul 17;15(7):e0235981. doi: 10.1371/journal.pone.0235981 (PMC7367453; doi:10.1371/journal.pone.0235981)

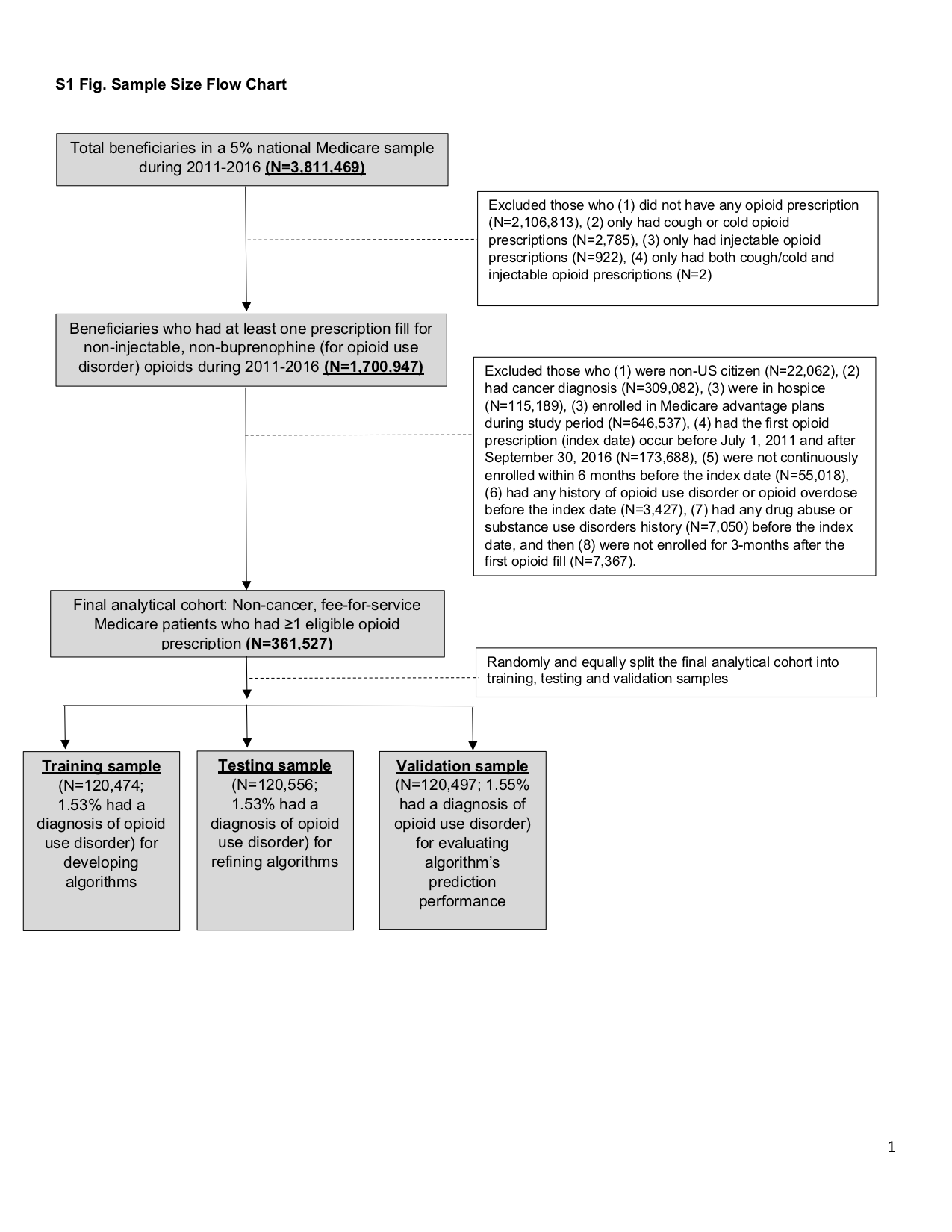

Supplement: S1 Fig — (TIF) [file pone.0235981.s011.tif]

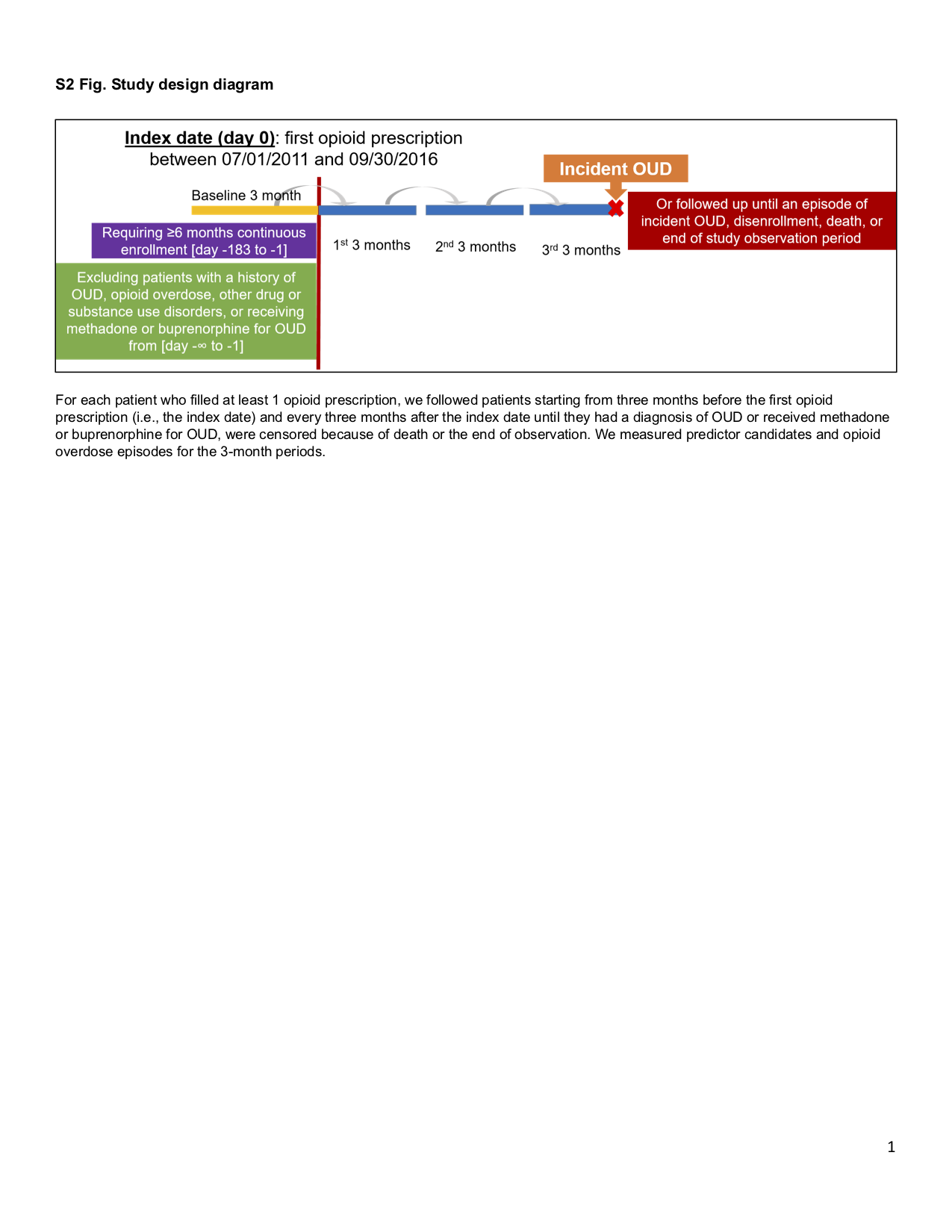

Supplement: S2 Fig — (TIF) [file pone.0235981.s012.tif]

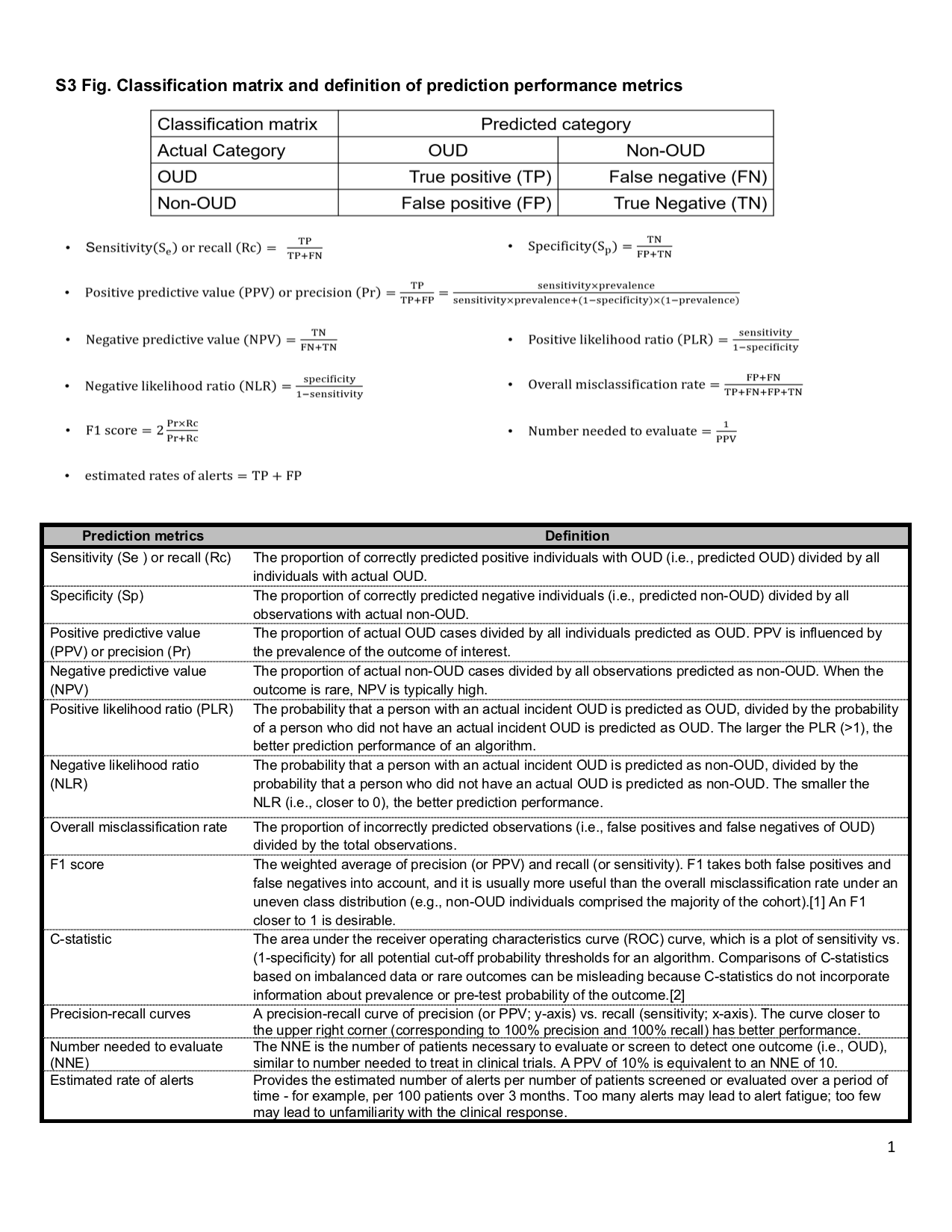

Supplement: S3 Fig — Saito T, Rehmsmeier M. The precision-recall plot is more informative than the ROC plot when evaluating binary classifiers on imbalanced datasets. PLoS One. 2015;10(3):e0118432. Epub 2015/03/05. doi: 10.1371/journal.pone.0118432. PubMed PMID: 25738806; PubMed Central PMCID: PMCPMC4349800. Romero-Brufau S, Huddleston JM, Escobar GJ, Liebow M. Why the C-statistic is not informative to evaluate early warning scores and what metrics to use. Crit Care. 2015;19:285. Epub 2015/08/14. doi: 10.1186/s13054-015-0999-1. PubMed PMID: 26268570; PubMed Central PMCID: PMCPMC4535737. (TIF) [file pone.0235981.s013.tif]

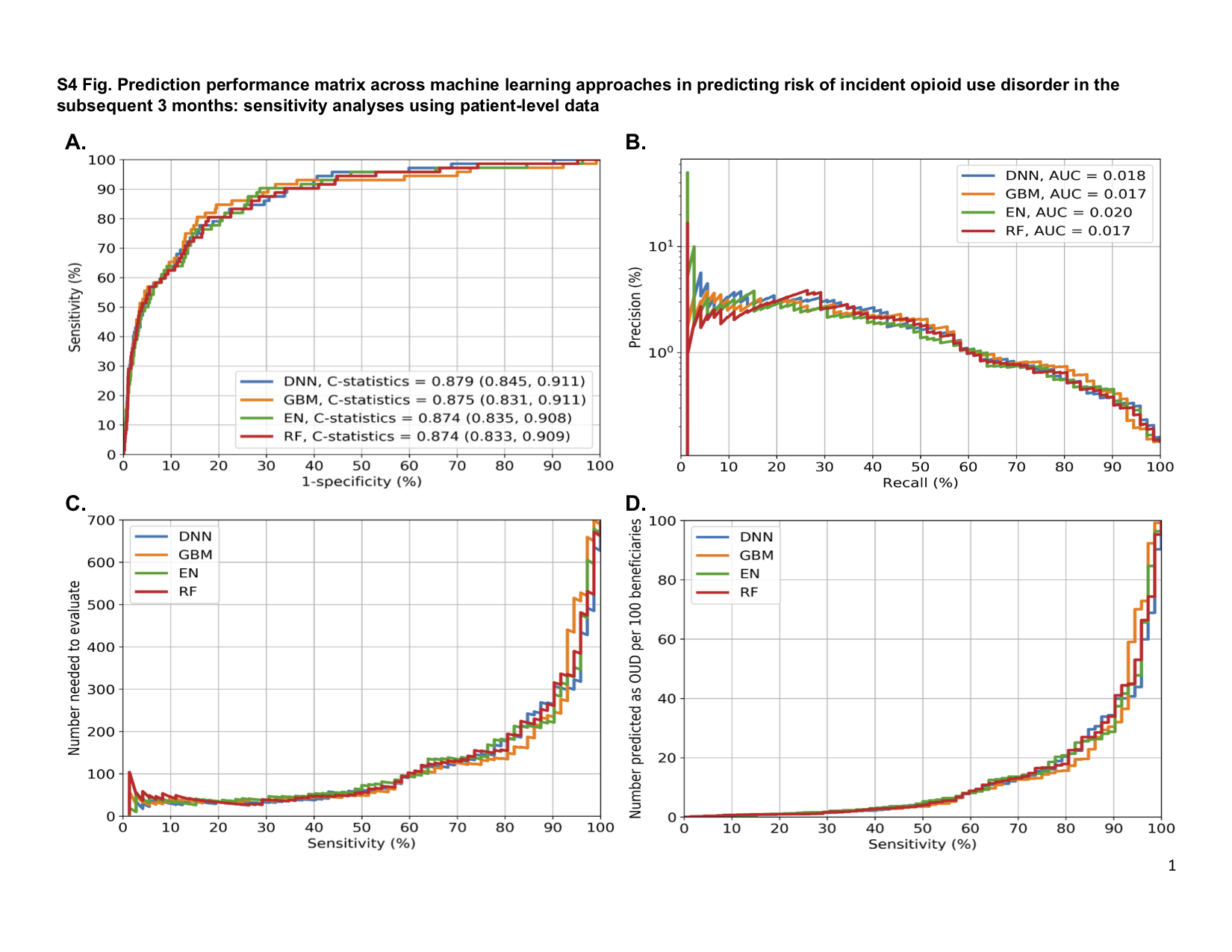

Supplement: S4 Fig — Figure shows four prediction performance matrices using an example of using randomly and iteratively selected patient-level data (n = 50,000 [49,927 non-OUD and 73 OUD patients], excluding those who had an OUD from the first 3-month period) from the validation sample. S4A Fig shows the areas under ROC curves (or C-statistics); S4B Fig shows the precision-recall curves (precision = PPV and recall = sensitivity)—precision recall curves that are closer to the upper right corner or above the other method have improved performance; S4C Fig shows the number needed to evaluate by different cutoffs of sensitivity; and S4D Fig shows alerts per 100 patients by different cutoffs of sensitivity. Abbreviations: AUC: area under the curves; DNN: deep neural network; EN: elastic net; GBM: gradient boosting machine; RF: random forest; ROC: Receiver Operating Characteristics. (TIF) [file pone.0235981.s014.tif]

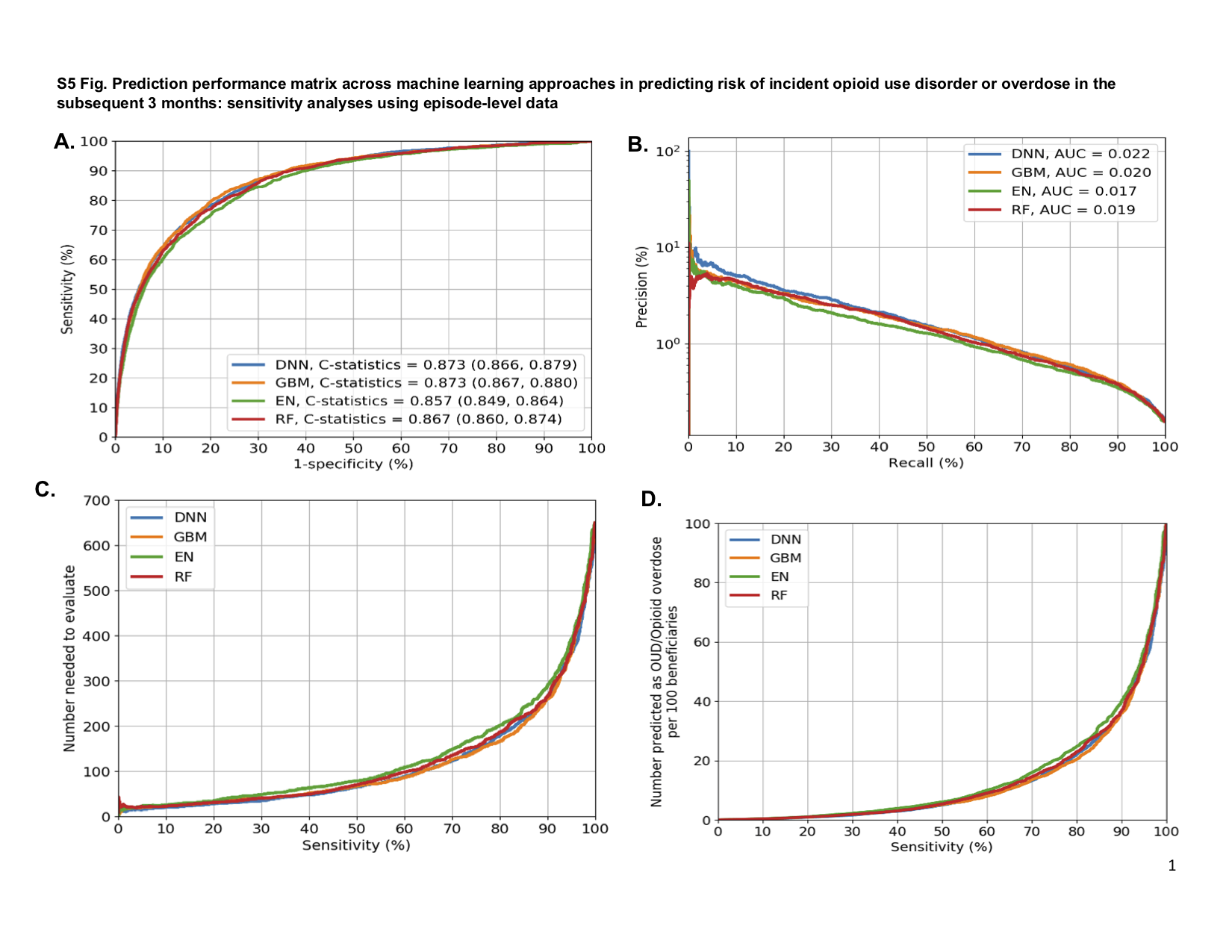

Supplement: S5 Fig — Figure shows four prediction performance matrices for predicting incident OUD or overdose in the subsequent three months at the episode level from the validation sample. S5A Fig shows the areas under ROC curves (or C-statistics); S5B Fig shows the precision-recall curves (precision = PPV and recall = sensitivity)—precision recall curves that are closer to the upper right corner or above the other method have improved performance; S5C Fig shows the number needed to evaluate by different cutoffs of sensitivity; and S5D Fig shows alerts per 100 patients by different cutoffs of sensitivity. Abbreviations: AUC: area under the curves; DNN: deep neural network; EN: elastic net; GBM: gradient boosting machine; OUD: opioid use disorder; RF: random forest; ROC: Receiver Operating Characteristics. (TIF) [file pone.0235981.s015.tif]

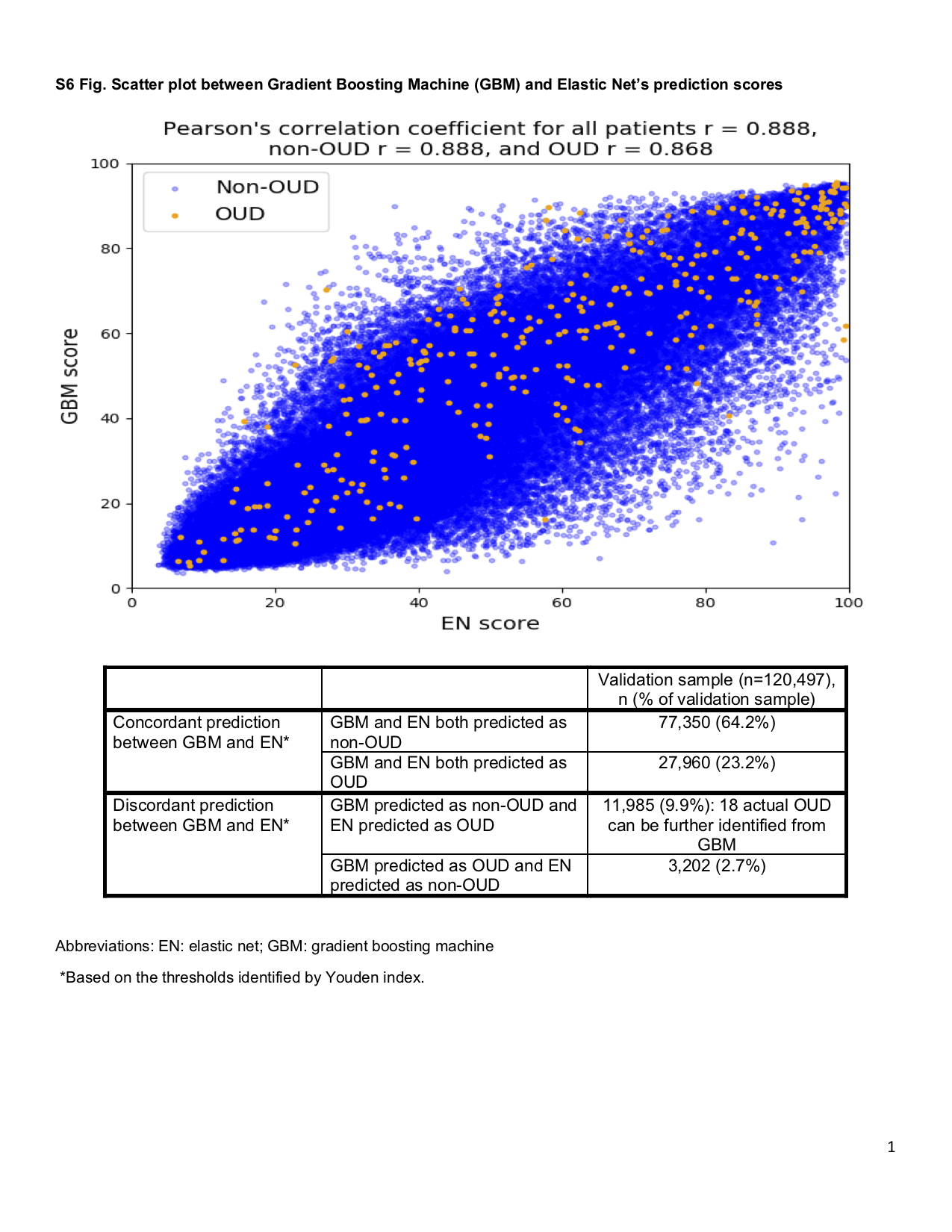

Supplement: S6 Fig — (TIF) [file pone.0235981.s016.tif]

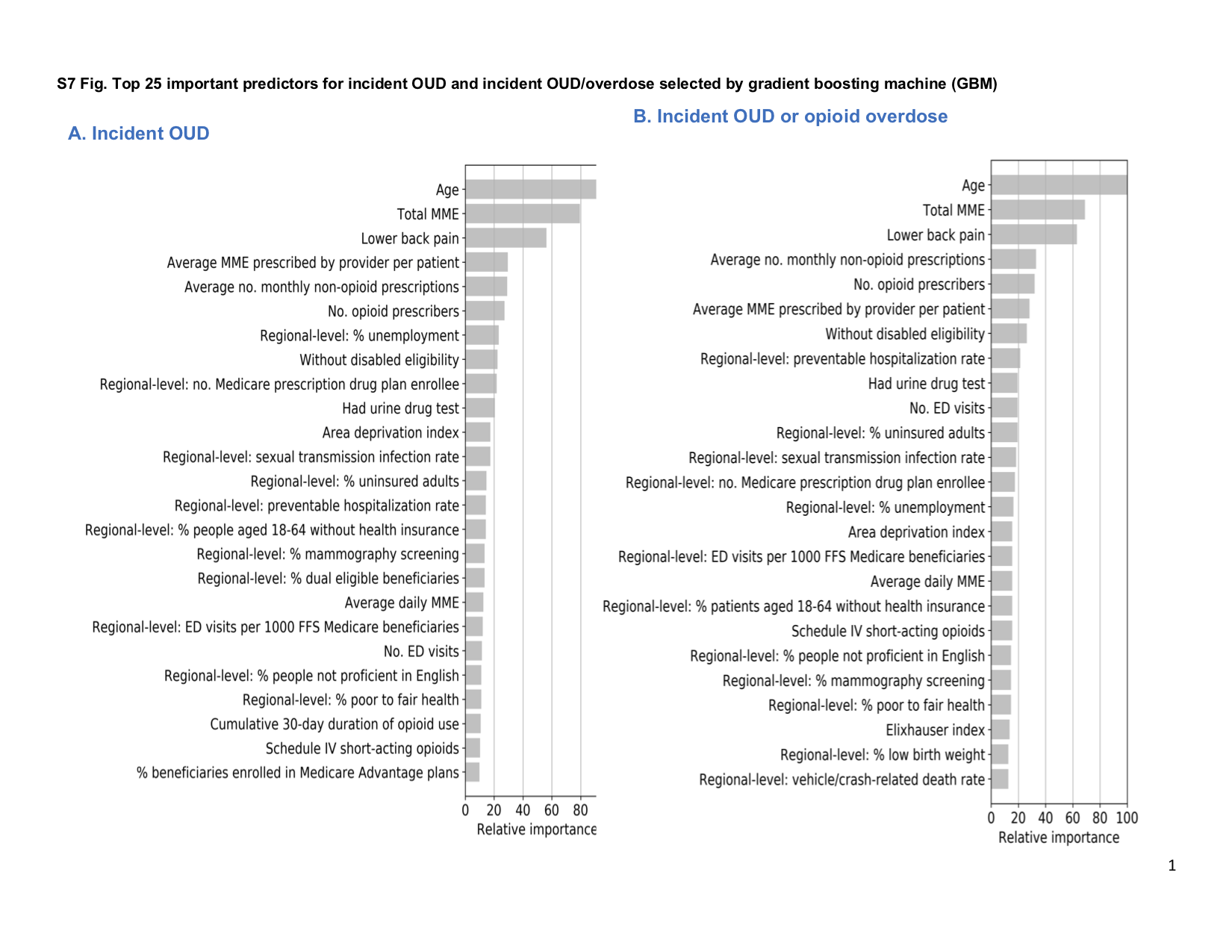

Supplement: S7 Fig — Abbreviations: ED: emergency department; FFS: fee-for-service; GBM: gradient boosting machine; MME: morphine milligram equivalent; No: number of a Rather than p values or coefficients, the GBM reports the importance of predictor variables included in a model. Importance is a measure of each variable’s cumulative contribution toward reducing square error, or heterogeneity within the subset, after the data set is sequentially split based on that variable. Thus, it is a reflection of a variable’s impact on prediction. Absolute importance is then scaled to give relative importance, with a maximum importance of 100. For example, the top 5 important predictors identified from GBM included age, total cumulative MME, lower back pain, average MME prescribed by provider per patient, and averaged no. monthly non-opioid prescriptions. (TIF) [file pone.0235981.s017.tif]

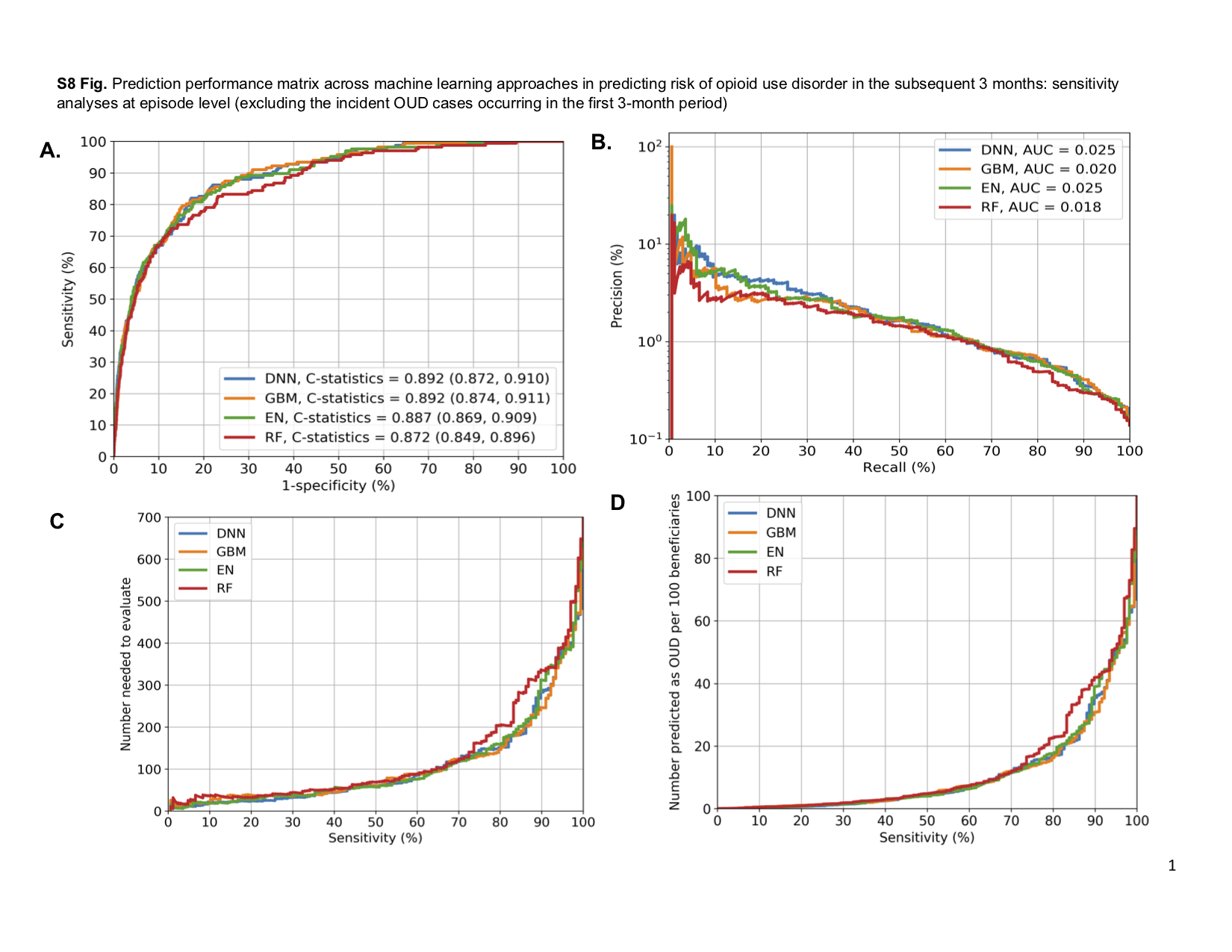

Supplement: S8 Fig — Figure shows four prediction performance matrices excluding opioid disorder outcomes occurred in the first 3 months after the index date in the validation sample. S8A Fig shows the areas under ROC curves (or C-statistics); S8B Fig shows the precision-recall curves (precision = PPV and recall = sensitivity)—precision recall curves that are closer to the upper right corner or above the other method have improved performance; S8C Fig shows the number needed to evaluate by different cutoffs of sensitivity; and S8D Fig shows alerts per 100 patients by different cutoffs of sensitivity. Abbreviations: AUC: area under the curves; DNN: deep neural network; EN: elastic net; GBM: gradient boosting machine; RF: random forest; ROC: Receiver Operating Characteristics. (TIF) [file pone.0235981.s018.tif]

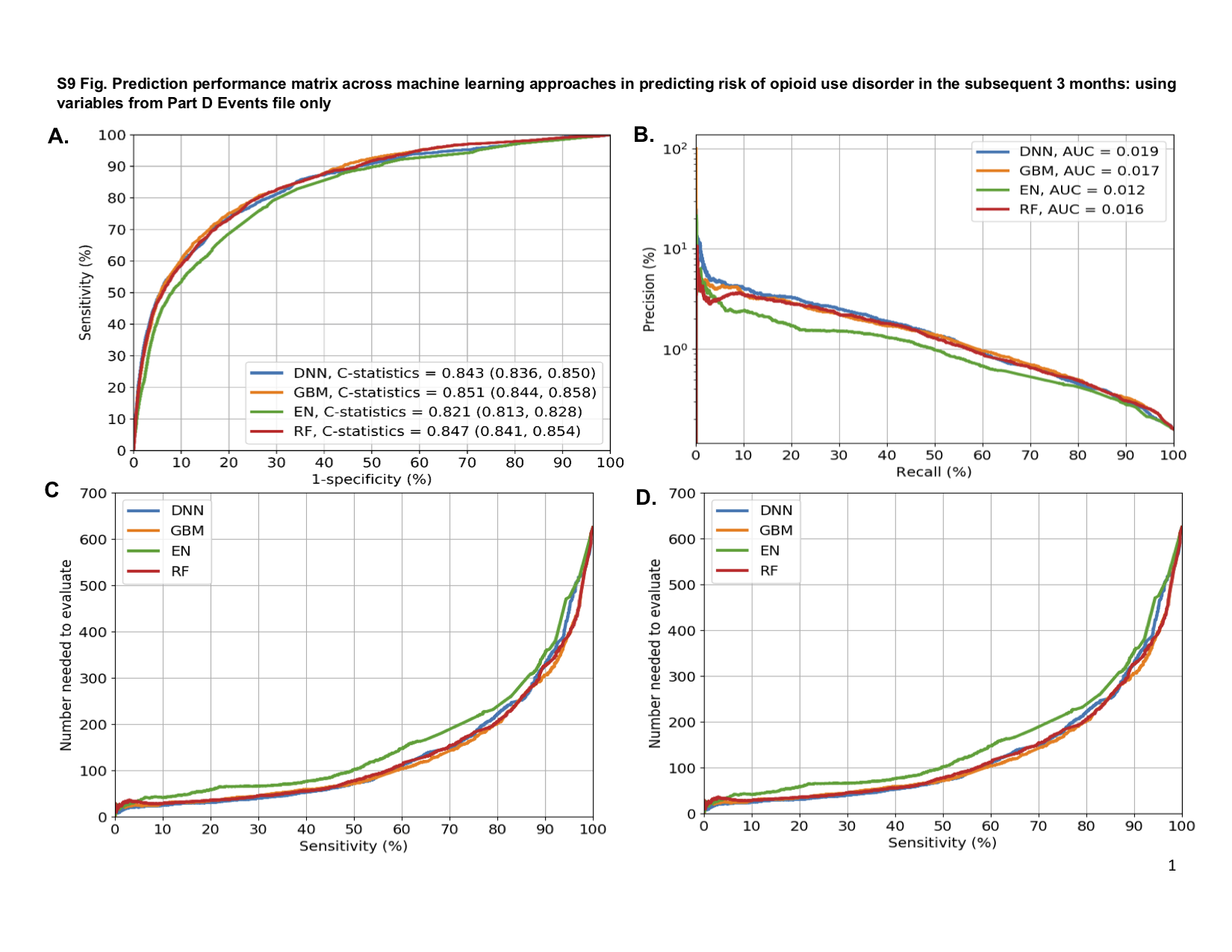

Supplement: S9 Fig — Figure shows four prediction performance matrices using only variables from Prescription Drug Events files in the validation sample. S9A Fig shows the areas under ROC curves (or C-statistics); S9B Fig shows the precision-recall curves (precision = PPV and recall = sensitivity)—precision recall curves that are closer to the upper right corner or above the other method have improved performance; S9C Fig shows the number needed to evaluate by different cutoffs of sensitivity; and S9D Fig shows alerts per 100 patients by different cutoffs of sensitivity. Abbreviations: AUC: area under the curves; DNN: deep neural network; EN: elastic net; GBM: gradient boosting machine; RF: random forest; ROC: Receiver Operating Characteristics. (TIF) [file pone.0235981.s019.tif]
