# Supplementary material for: Using machine learning to predict risk of incident opioid use disorder among fee-for-service Medicare beneficiaries: A prognostic study
Source: PLoS One. 2020 Jul 17;15(7):e0235981. doi: 10.1371/journal.pone.0235981 (PMC7367453; doi:10.1371/journal.pone.0235981)
Supplement: Attachment — Submitted filename: 20200526_Response to Reviewers_v3_clean.docx [file pone.0235981.s020.docx]

**Journal Requirements:** When submitting your revision, we need you to address these additional requirements.

1. Please ensure that your manuscript meets PLOS ONE's style requirements, including those for file naming. The PLOS ONE style templates can be found at <https://journals.plos.org/plosone/s/file?id=wjVg/PLOSOne_formatting_sample_main_body.pdf> and <https://journals.plos.org/plosone/s/file?id=ba62/PLOSOne_formatting_sample_title_authors_affiliations.pdf>
   - **We have reformatted and revised our manuscript to ensure it meets PLOS ONE’s requirements.**
2. We noticed you have some minor occurrence of overlapping text with the following previous publication(s), which needs to be addressed: <https://jamanetwork.com/journals/jamanetworkopen/fullarticle/2728625>
   [https://onlinelibrary.wiley.com/doi/10.1002/pds.4864](https://urldefense.proofpoint.com/v2/url?u=https-3A__onlinelibrary.wiley.com_doi_10.1002_pds.4864&d=DwQGaQ&c=sJ6xIWYx-zLMB3EPkvcnVg&r=pDlPdDBMHMsG2D-njUbaUVX-AZD70Bprw27QJ6CMgdc&m=0-IvrP3aCRwbYHypxnwUkadLHFahoAkgXM_9sVI5cKw&s=x8PZeDGMqXB09ziYG21JsVTGh02uAf8BoBm3ZmMBOl0&e=). In your revision ensure you cite all your sources (including your own works), and quote or rephrase any duplicated text outside the methods section. Further consideration is dependent on these concerns being addressed.
   - **We thoroughly checked the text in our manuscript and have now rephrased texts duplicated outside the method section in our JAMA Network Open work in our revision.**
3. We note that you have indicated that data from this study are available upon request. PLOS only allows data to be available upon request if there are legal or ethical restrictions on sharing data publicly. For information on unacceptable data access restrictions, please see [http://journals.plos.org/plosone/s/data-availability#loc-unacceptable-data-access-restrictions](https://urldefense.proofpoint.com/v2/url?u=http-3A__journals.plos.org_plosone_s_data-2Davailability-23loc-2Dunacceptable-2Ddata-2Daccess-2Drestrictions&d=DwQGaQ&c=sJ6xIWYx-zLMB3EPkvcnVg&r=pDlPdDBMHMsG2D-njUbaUVX-AZD70Bprw27QJ6CMgdc&m=0-IvrP3aCRwbYHypxnwUkadLHFahoAkgXM_9sVI5cKw&s=Vo8uEL-zai3fzrPjOKwi2mPeRgxZUqgz5wW6roIWHHM&e=).

   In your revised cover letter, please address the following prompts:

   a) If there are ethical or legal restrictions on sharing a de-identified data set, please explain them in detail (e.g., data contain potentially identifying or sensitive patient information) and who has imposed them (e.g., an ethics committee). Please also provide contact information for a data access committee, ethics committee, or other institutional body to which data requests may be sent.

   b) If there are no restrictions, please upload the minimal anonymized data set necessary to replicate your study findings as either Supporting Information files or to a stable, public repository and provide us with the relevant URLs, DOIs, or accession numbers. Please see [http://www.bmj.com/content/340/bmj.c181.long](https://urldefense.proofpoint.com/v2/url?u=http-3A__www.bmj.com_content_340_bmj.c181.long&d=DwQGaQ&c=sJ6xIWYx-zLMB3EPkvcnVg&r=pDlPdDBMHMsG2D-njUbaUVX-AZD70Bprw27QJ6CMgdc&m=0-IvrP3aCRwbYHypxnwUkadLHFahoAkgXM_9sVI5cKw&s=9TZjD5zr9f2t2D7DIKIdYlv2kFiwU3Ha8Adi6iPtMo8&e=) for guidelines on how to de-identify and prepare clinical data for publication. For a list of acceptable repositories, please see [http://journals.plos.org/plosone/s/data-availability#loc-recommended-repositories](https://urldefense.proofpoint.com/v2/url?u=http-3A__journals.plos.org_plosone_s_data-2Davailability-23loc-2Drecommended-2Drepositories&d=DwQGaQ&c=sJ6xIWYx-zLMB3EPkvcnVg&r=pDlPdDBMHMsG2D-njUbaUVX-AZD70Bprw27QJ6CMgdc&m=0-IvrP3aCRwbYHypxnwUkadLHFahoAkgXM_9sVI5cKw&s=dKOd3RUorvU05V-pNfrWLRR1p-TBoa98-97CeZJ_w6k&e=).

   We will update your Data Availability statement on your behalf to reflect the information you provide.

- **We updated our statement on data sharing and data availability after checking our data use agreement with the Centers for Medicare and Medicaid Services (CMS).**

**“Data availability: Data are available from the Centers for Medicare and Medicaid Services for a fee and under data use agreement provisions. Per the data use agreement, the relevant limited data sets cannot be made publicly available. The website’s reference on how others may access the relevant data, in the same manner as it was accessed by the authors of this study, is** [**https://www.resdac.org/cms-virtual-research-data-center-vrdc-faqs**](https://www.resdac.org/cms-virtual-research-data-center-vrdc-faqs)**.”**

4. Thank you for stating the following in the Competing Interests section:
   "I have read the journal's policy and the authors of this manuscript have the following
   competing interests: Dr. Kwoh has received honoraria from AbbVie and EMD Serono and has provided consulting services for Astellas, Thusane, and Novartis, EMD Serono and Express Scripts."

   Please confirm that this does not alter your adherence to all PLOS ONE policies on sharing data and materials, by including the following statement: "This does not alter our adherence to PLOS ONE policies on sharing data and materials.” (as detailed online in our guide for authors [http://journals.plos.org/plosone/s/competing-interests](about:blank)).  If there are restrictions on sharing of data and/or materials, please state these. Please note that we cannot proceed with consideration of your article until this information has been declared.

   Please include your updated Competing Interests statement in your cover letter; we will change the online submission form on your behalf.

   Please know it is PLOS ONE policy for corresponding authors to declare, on behalf of all authors, all potential competing interests for the purposes of transparency. PLOS defines a competing interest as anything that interferes with, or could reasonably be perceived as interfering with, the full and objective presentation, peer review, editorial decision-making, or publication of research or non-research articles submitted to one of the journals. Competing interests can be financial or non-financial, professional, or personal. Competing interests can arise in relationship to an organization or another person. Please follow this link to our website for more details on competing interests: [http://journals.plos.org/plosone/s/competing-interests](about:blank)
   - **We have checked with all of the coauthors and updated the statement regarding their competing interests in our cover letter. We confirm that “this does not alter our adherence to PLOS ONE policies on sharing data and materials.”**
5. Please include captions for your Supporting Information files at the end of your manuscript, and update any in-text citations to match accordingly. Please see our Supporting Information guidelines for more information: [http://journals.plos.org/plosone/s/supporting-information](about:blank).
   - **We now include captions for our Supporting information files at the end of our revised manuscript. We carefully matched them to correspond with our in-text citations.**

**Reviewers' comments:**

**Reviewer #1:**

Major comments:

6. The clinical utilization of the algorithm is not clear. The authors have identified 25 important predictors. However, it is not clear how to use these predictors in clinical practice.

- **The primary clinical utility of our machine-learning algorithm is to create a prediction risk probability or risk score for developing an incident OUD in the subsequent 3 months. Our intention in showing the top 25 important predictors was to provide some insights of variables relevant for prediction because some machine learning methods (e.g., DNN) have been criticized as black-box approaches. However,** **interpreting individual important predictors separately should be done cautiously. We added sentences to clarify this in the Methods.**

**Page 10 (lines 188-192): “To increase clinical utility, we conducted several additional analyses. First, while the primary clinical utility of our machine-learning algorithm is to create a prediction risk score for developing incident OUD, we report the top 25 important predictors to provide some insights on variables relevant for prediction. However, interpreting individual important predictors separately or for causal inference should be done cautiously.”**

Minor comments

7. Figure 3: What does “e.g.” indicate? Different states have different OR?
   - **We modified the label as “Resided in Nevada” because residing in the state of Nevada was the only indicator selected as an important predictor among all of the state residency indicators.**

**Reviewer #2:**

8. Page 4. Abstract. “CONCLUSIONS: Machine-learning algorithms improve risk prediction and stratification of incident OUD, especially in identifying low-risk subgroups that have negligible risk.”
   The main objective of this study is to develop a machine-learning algorithm which could help identify patients at OUD risks, while the statement regarding the low-risk groups may not be directly related to the main purpose.
   - **The objective of this study is to develop and validate a machine-learning algorithm to improve risk prediction and stratification of incident OUD diagnosis. Therefore, our machine learning algorithm may help rule out individuals with minimal risk from those with higher risk in the context of rare outcomes. We modified our Conclusions in the Abstract as “Machine-learning algorithms improve risk prediction and risk stratification of incident OUD in Medicare beneficiaries.”**
9. Page 15. Line 271. “Figure 3 shows the 25 most important predictors identified by EN, including lower back pain,”
   It would be great to discuss the clinical implications for these predictors, and potential interventions. Patients with lower back pain are very likely to receive opioids, would that be considered as OUD?
   - **Please see our response to Comment #6. Lower back pain is only one of the important factors contributing to the incident OUD prediction. An individual’s overall risk score for incident OUD needs to be considered for a complete picture of the selected important predictors, and interpreting individual predictors for causal inference may not be appropriate.**
10. Page 19. Line 337. “determine whether a patient is at high risk of incident OUD compared to current CMS”
    The data for this study was up to 2016, is there any changes in the policy or physician’s practices? Would it be necessary to update the algorism using most recent data?
    - **We added a limitation to address the potential need to update our algorithms as opioid-related policies and practice have changed over time.**

**“Furthermore, our study used publicly available older data. Updating and refining the prediction algorithm on a regular basis (e.g., quarterly or yearly) is recommended as opioid-related policies and practices have changed over time.”**
